# New Unsymmetrically Substituted Benzothiadiazole-Based Luminophores: Synthesis, Optical, Electrochemical Studies, Charge Transport, and Electroluminescent Characteristics

**DOI:** 10.3390/molecules26247596

**Published:** 2021-12-15

**Authors:** Pavel S. Gribanov, Dmitry A. Loginov, Dmitry A. Lypenko, Artem V. Dmitriev, Sergey I. Pozin, Alexey E. Aleksandrov, Alexey R. Tameev, Igor L. Martynov, Andrey Yu. Chernyadyev, Sergey N. Osipov

**Affiliations:** 1 A. N. Nesmeyanov Institute of Organoelement Compounds, Russian Academy of Sciences, 28 Vavilova Str., 119991 Moscow, Russia; gribanovps@mail.ru (P.S.G.); dloginov@ineos.ac.ru (D.A.L.); 2 A. N. Frumkin Institute of Physical Chemistry and Electrochemistry, Russian Academy of Sciences, Leninsky Prosp. 31, bld.4, 119071 Moscow, Russia; dalypenko@gmail.com (D.A.L.); oleduff@mail.ru (A.V.D.); sergip74@gmail.com (S.I.P.); klays007@gmail.com (A.E.A.); tameev@elchem.ac.ru (A.R.T.); chernyadyev@mail.ru (A.Y.C.); 3 Institute for Nanoengineering in Electronics, Spintronics and Photonics, National Research Nuclear University “MEPhI”, Kashirskoe Shosse 31, 115409 Moscow, Russia; i.l.martynov@gmail.com

**Keywords:** luminophore, benzothiadiazole, cross-coupling, luminescence, phosphorescence, OLED

## Abstract

Three new benzothiadiazole (BTD)-containing luminophores with different configurations of aryl linkers have been prepared via Pd-catalyzed cross-coupling Suzuki and Buchwald–Hartwig reactions. Photophysical and electroluminescent properties of the compounds were investigated to estimate their potential for optoelectronic applications. All synthesized structures have sufficiently high quantum yields in film. The BTD with aryl bridged carbazole unit demonstrated the highest electrons and holes mobility in a series. OLED with light-emitting layer (EML) based on this compound exhibited the highest brightness, as well as current and luminous efficiency. The synthesized compounds are not only luminophores with a high photoluminescence quantum yield, but also active transport centers for charge carriers in EML of OLED devices.

## 1. Introduction

The studies on covalently linked donor–acceptor (D-A) molecular systems have attracted considerable interest in different organic electronic applications [1,2,3,4,5,6,7,8,9,10,11,12,13,14,15,16,17,18,19,20,21]. The use of appropriately positioned, easy to oxidize donor and easy to reduce acceptor molecules promotes the possibility of D-to-A charge transfer extending their absorption well into the visible and near-IR regions that can be exploited in organic optoelectronic devices. In recent years the 2,1,3-benzothiadiazole (BTD) as a good acceptor unit installed in the multimodular D-A systems has gained significant attention due to its several advantages such as favorable reduction potential, a prominent bathochromic shift of the charge transfer absorption band, and strong electron affinity. The BTD derivatives have widely been used as π-conjugated organic materials for two-photon absorption, photoinduced intramolecular charge transfer (ICT), organic light-emitting diodes (OLED), and solar cells [4,22,23,24,25,26,27,28,29]. The nature of the donor and of the acceptor and the geometry of the linker between them are important factors that affect the properties of such compounds. The introduction of rigid chromophores with twisted molecular geometry or alkyl substituents into aromatic linker can prevent unfavorable π-aggregates in solid state, which often cause fluorescence quenching. On the other hand, the moderate π-π interaction that is inherent to non-planar D-A systems, has been shown is necessary for better charge hopping in the devices [30,31,32].

**Figure 1:**
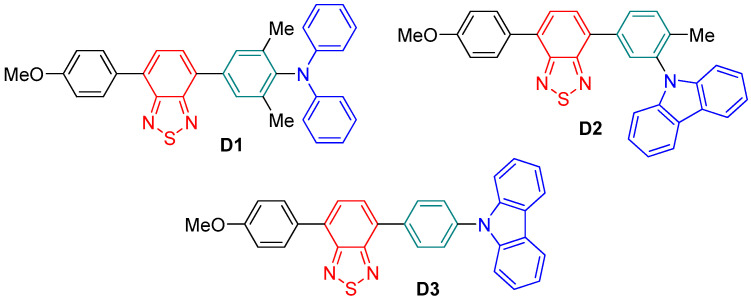
Structures of the BTD Dyes **D1**, **D2**, **D3**.

Taking into account the facts mentioned above and in continuation of our research in the field [33], here we wish to disclose an efficient synthesis of three new luminophores with different configuration of aryl linker (Figure 1), their photo-physical properties and the working characteristics of OLEDs fabricated on their basis as well.

## 2. Results and Discussion 

### 2.1. Synthesis

**Scheme 1:**
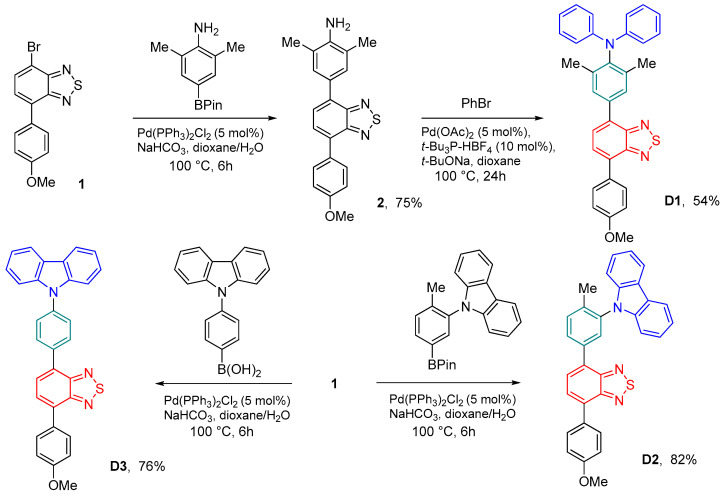
Synthesis of dyes **D1**, **D2**, **D3**.

A synthetic route to the BTDs **D1** included a sequence of two Pd-catalyzed cross-coupling reactions starting from readily available 4-bromosubstituted BTD **1** [33,34,35,36] (Scheme 1). The first Suzuki coupling of **1** with 2,6-dimethyl-4-(4,4,5,5-tetramethyl-1,3,2-dioxaborolan-2-yl)aniline [37] was accomplished by slightly modified procedure previously described for selective formation of unsymmetrical BTD molecules [33].

For the next double Buchwald–Hartwig coupling of **2** with bromobenzene the combination of 5 mol % Pd(OAc)_2_, 10 mol % *t*-Bu_3_P-HBF_4_ and 2.4 equiv. *t*-BuONa has proved to be the most effective catalytic system among tested to furnish the desired **D1** in good yield. In the case of **D2** the corresponding cross-coupling partner, 9-(2-methyl-5-(4,4,5,5-tetramethyl-1,3,2-dioxaborolan-2-yl)phenyl)-9H-carbazole, has been specially synthesized via two step procedure (see Experimental Section). The final compounds **D2**, **D3** have been successfully obtained using the same Suzuki conditions as for **2**. All compounds **D1**, **D2**, **D3** were isolated by flash chromatography on silica gel and additionally purified by sublimation (200–220 °C/0.1 Torr). Their structures were characterized using ^1^H NMR, ^13^C NMR, and HRMS (see the Supplementary Materials).

### 2.2. Optical Properties

It is well known that the introduction of an electron-donor amino group at position 4 of 2,1,3-benzothiadiazole significantly changes its absorption spectrum in the near UV region: a broad low-energy band appears in the region of 400 nm [36,38]. The absorption spectra of compounds **D1**, **D2**, **D3** in *m*-xylene solution are shown in Figure 1. A broad maximum in the region of 410 nm at the long-wavelength edge of the spectrum (see Table 1) corresponds to charge transfer (CT) between the donor (carbazole or diphenylamine) and acceptor (benzothiadiazole) fragments (D and A). The higher energy band in the absorption spectra corresponds to the typical electronic transition of molecules. For the carbazole-containing molecules, this band exhibits characteristic narrow maxima corresponding to carbazole moiety.

The localization of the frontier molecular orbitals as well as spectral assignments of low-energy absorption bands were estimated from DFT and TD-DFT calculations (see the Supplementary Materials). Indeed, in all compounds **D1**, **D2**, **D3**, the LUMO orbital is expectedly located on the benzothiadiazole fragment (Table S1). However, the HOMO orbital is mainly located on the donor moieties (carbazole or diphenylamine) with different partial delocalization on the benzothiadiazole and aryl linker depending on the dihedral angles between cyclic frames. For example, compound **D2** with high dihedral angle between carbazole and aryl linker (ca. 80°, see Table S2) has almost separated HOMO and LUMO orbitals, which leads to the absence of π-conjugation between D and A. Therefore, the low-energy absorption band for **D2** corresponds to the π → π* transition, which is formed by HOMO-1 → LUMO orbitals (Table S3). At the same time, the dihedral angles in **D1** and **D3** do not exceed 55°, which provides partial delocalization of HOMO on aryl linker for **D1** or on all remaining fragments for **D3**. The partial delocalization in these cases leads to inclusion of CT (HOMO → LUMO transition) to the low-energy absorption bands. It should be noted that aryl linker plays an important role in delocalization of HOMO orbital. For example, no π-conjugation between D and A has been observed in closely related phenoxazine and phenothiazine compounds without aryl linker between D and A [33].

**Figure 2:**
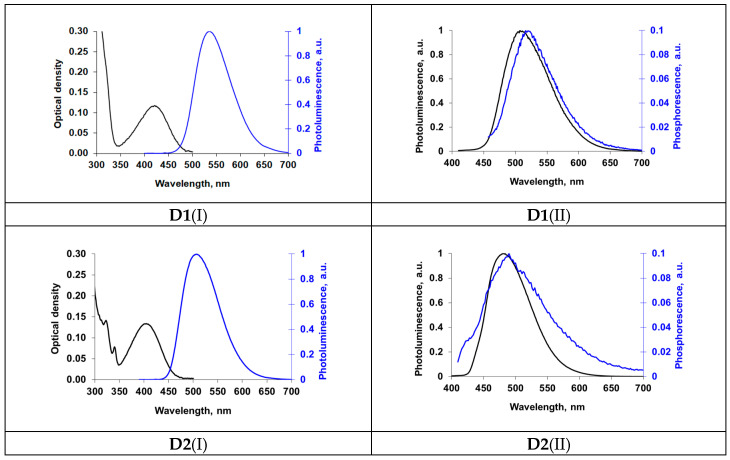

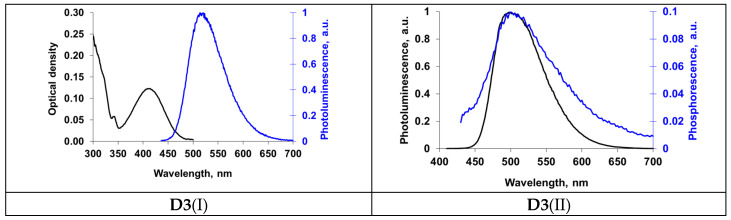
Optical spectra of **D1**, **D2**, **D3** in *m*-xylene (concentration 10^−5^ M, optical length 1 cm, λ_ex_ = 375–400 nm). (**I**) Absorption and photoluminescence at room temperature; (**II**) photoluminescence and phosphorescence (delay 1 ms) at 77 K.

The CT bands in the absorption spectra of **D1**, **D2**, **D3** are mirror-symmetrical to their broad emission bands in the photoluminescence spectra (PL) spectra at 298 K (see Figure 2, Table 1). These emission bands are characterized by a high quantum yield in non-polar solvents, a significant Stokes shift, and a positive solvatochromic effect (up to 700 cm^−1^ when *m*-xylene is replaced with chlorobenzene, see the Supplementary Materials). It was reported that for some compounds similar to **D1** with a rigid molecule framework and without a phenyl bridge between the amine donor and the benzothiadiazole acceptor, the dependence of PL spectra on the excitation wavelength in non-polar solvent has been revealed [36]. However, in the case of compounds **D1**, **D2**, **D3** such dependence was not found. The position of the PL maximum and the values of the PL quantum yields for films of these compounds (when co-deposited with the mCP matrix (1,3-bis(*N*-carbazolyl)benzene), 10 wt %, 298 K) are also given in Table 1. For compounds with a carbazole donor (**D2**, **D3**), in contrast to **D1**, the PL maximum in the mCP matrix underwent a noticeable bathochromic shift compared to the PL maximum in *m*-xylene solution. The difference in the quantum yields for **D2** and **D3**, presumably, could be explained by the different position of the carbazole substituent (*para*- or *meta*-position relative to the acceptor); examples of such an effect of substituents in the benzene ring are known [39,40].

At 77 K, the PL bands are hypsochromically shifted (see Figure 2, Table 1), and mirror symmetry is broken due to the manifestation of phosphorescence bands (a weak shoulder appears in the PL spectra to the right of the main maximum). Phosphorescence (P) of compounds **D1**–**3** with a delay of 1 ms to disable prompt fluorescence is also shown in Figure 1. Compound **D1** with a triphenylamine fragment demonstrates the best ability to phosphorescence. Thus, P lifetime for **D2** and **D3** was about 2 ms, whereas for **D1** it was 200 ms. The maxima of the P bands are shifted relative to the corresponding PL maxima by 5 nm, approximately, for compounds with a carbazole donor and by 10 nm for compounds with a triphenylamine donor. The P bands are narrowed in comparison with the PL bands (this is noticeable by 3a in Figure 1, for which the spectra were recorded with the same bandpass value). The observed shift of the P maxima relative to the PL maxima at 77 K in energy scale corresponds to: 0.02–0.03 eV for **D2**, **D3** (close to the thermal energy at 298 K), and 0.06 eV for **D1**. These values are not accurate ∆E_ST_ due to the possible difference in the coordinates of the molecule nucleus in the singlet and triplet excited states [41], as well as that the observed P bands at 77K may be a superposition of different phosphorescence: from CT state or from locally excited (LE) state of any part of the molecules [42,43].

**Figure 3:**
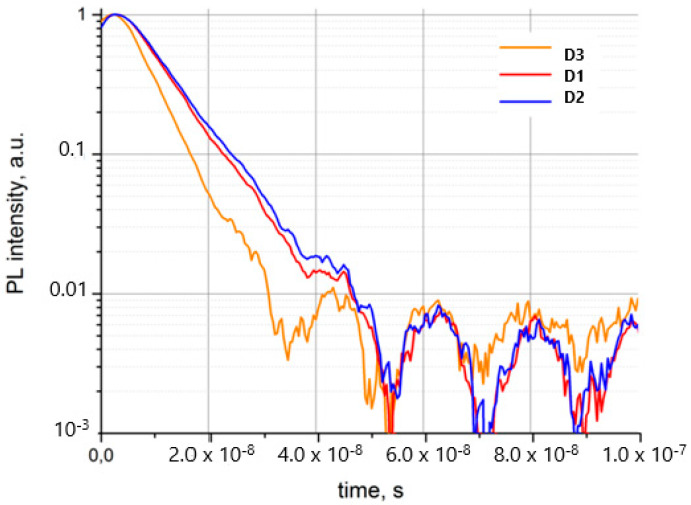
Photoluminescence decay curves of **D1**, **D2**, **D3** in semilogarithmic scale. λ_ex_ = 355 nm, λ_em_ = 550 nm.

The presence of a CT-state in the molecule and small values of ∆E_ST_ are necessary but not sufficient for the occurrence of effective delayed *E*-type fluorescence (TADF) [24,44,45,46]. To elucidate the presence of the TADF effect for the synthesized compounds, photoluminescence decay curves were recorded in the nano- and microsecond range upon excitation of the sample with a laser pulse diode (Figure 3). Samples of films on a quartz substrate (**D1**, **D2**, **D3** in mCP matrix, 10 wt %) were investigated at 298 K. TXO-PhCz (compound with a carbazole donor and thioxanthone acceptor [45]) was used as a control sample. The decay curves for **D1**, **D2**, **D3** are linear in semilogarithmic coordinates; the luminescence lifetime was ~5 ns for **D3**, and ~10 ns for **D1**, **D2**. The behavior of control sample was in accordance with the published data [45]. Two sections can be distinguished on its curve in the range of 1 ms with times of ~0.2 μs and ~50 μs in addition to the prompt fluorescence. The decay curves for **D1**, **D2**, **D3** recorded under the same conditions do not contain any other components besides the prompt fluorescence. Therefore, it can be assumed that the compounds obtained do not exhibit the TADF effect under these conditions. Moreover, TD-DFT calculations (Table S2) showed that energy gap between T_1_ and S_1_ excitation states of compounds **D1**, **D2**, **D3** is very high (0.5–0.8 eV), which prevents reverse intersystem crossing process from T_1_ to S_1_. At the same time, all compounds exhibit rather small energy gaps between T_2_ and S_1_ (0.02–0.26 eV) that allow us to consider these compounds as perspective hybridized local and charge-transfer excited-state fluorophores, which can enable full exciton utilization through a reverse intersystem crossing from high-lying triplet states to singlet state [40].

In general, we state that in the photophysical aspect compounds **D1**, **D2**, **D3** are similar to their symmetrical analogs described in [40]. In all cases, there is the D-π-A fragment with a phenyl bridge. A similar behavior can be noted in low-molecular carbazole-substituted matrices, namely, the presence of bathochromic shifts for compounds with a donor in the *para*-position, a strong dependence of quantum yields on the position of the donor and nanosecond values of the luminescence lifetime. The presence of phenyl bridges between the D and A facilitates conformational changes in the molecule. On the one hand, this leads to the fact that we do not observe the complex effects described for the bridge-free compounds with similar D-A blocks [36]. On the other hand, in the case of presence of the phenyl bridges in molecule, fine tuning of rotation angles between the D and A is required to obtain the TADF effect [47].

### 2.3. The Charge Transport Study

In the composite layers **D1**–**3/**mCP, the mCP served as a wide-gap matrix with HOMO and LUMO levels of −5.9 and −2.4 eV, respectively [48]. The charge carrier mobility measured by using the technique of charge extraction by linearly increasing voltage (CELIV) in layers of the pristine mCP and **D1**–**3/**mCP composites are listed in Table 2. The mobility of holes and electrons in the mCP layers are in a good agreement with those measured by the time-of-flight technique [49]. Indeed, both techniques are based on the measurement of transient currents caused by the transport of charge carriers with capture in traps.

The electron mobility, whose hopping transport proceeds over LUMO levels, is significantly higher in all **D1**–**3/**mCP composites than in the pure mCP layer. For example, the mobility in **D3/**mCP is higher by an order of magnitude than in the mCP matrix (Table 2). This is due to the fact that compounds **D1**, **D2**, **D3** contain a benzothiadiazole fragment, which is known as an effective electron transport center [32,50,51,52,53]. Due to the high concentration of **D1**–**3** (10 wt %) in the composite, charge transport centers of both the matrix and additives participate in the electron transport. The average distance between neighboring dopant molecules in composites less than 2 nm, estimated for uniformly distribution [54], is in good agreement with the hopping length of charge carriers. Moreover, the LUMO levels of **D1**, **D2**, **D3** are 0.8–1.0 eV lower than those of mCP, so there are no electron traps in the matrix.

The hole mobility in the **D2** and **D3** composites is higher than in a pure mCP layer. The compounds contain an electron-donor carbazole fragment; therefore, the position of their HOMO levels is close to the position of the corresponding levels of carbazole units in the mCP. In the case of the compound **D3**, the transfer of holes between its molecules proceeds without capture on the matrix mCP molecules, since its HOMO level is 0.215 eV higher than that of the mCP molecule. This explains the highest value of hole mobility of 4 × 10^−3^ cm^2^V^−1^s^−1^ compared with other studied materials. At the same time, the HOMO level of the mCP is 0.07 eV above the corresponding level of the compound **D2** so the mCP molecules can serve as shallow traps for holes during the hopping transport between **D2** molecules. For this reason, the hole mobility in the **D2**/mCP composite layer is only about 2 times higher than in the pure mCP layer.

The structure of the compound **D1** contain a biphenylamine substituent instead of a carbazole. The **D1** HOMO level is about 0.66 eV above the mCP HOMO level, so the matrix cannot capture holes during their transport between **D1** molecules. On the contrary, the **D1** molecules act as deep traps for holes that move in the mCP matrix, and therefore the mobility in the composite is lower than in the pure mCP matrix.

Thus, electron-donor and electron-acceptor moieties in the **D1**, **D2**, **D3** molecules provide the ambipolar conductivity of the composite layers. Compounds **D2** and **D3** which include both benzothiadiazole and carbazole fragments, increase the mobility of both electrons and holes in the mCP-based composite. For the **D3**/mCP composite, the mobility increase by an order of magnitude compared to that in the initial mCP matrix.

### 2.4. OLED Device Fabrication and Study of Their Spectral and Optoelectronic Properties

Compounds **D1**, **D2**, and **D3** have high quantum yields of luminescence (Table 1) and provides a relatively high mobility of charge carriers in composite layers (Table 2). They are also thermally stabile and are able to form amorphous films under deposition by thermal vacuum evaporation (TVE). Therefore, they are good candidates for use in OLED light-emitting layers. The EL properties of **D1**–**3/**mCP composites as light-emitting layers were investigated in a series of OLED devices: ITO/TAPC (60 nm)/**D1**–**3** (5–15 wt %)/mCP (25 nm)/TPYMB (30 nm)/LiF (1 nm)/Al (80 nm Here ITO is the anode, LiF/Al is the cathode and TAPC (1,1-bis[(di-4-tolylamino) phenyl] cyclohexane) is a hole transport layer (HTL) widely used in OLEDs [55]. In addition, TAPC can block electrons due to the high LUMO level (−2.0 eV). An electronic transport layer (ETL) was formed from tris-(2,4,6-trimethyl-3-(pyridin-3-yl)phenyl) borane (3TPYMB), which also serves as a hole blocking layer due to its wide band gap [56]. A light emitting layer (EML) **D1**–**3/**mCP was formed by co-deposition of **D1**–**3** and mCP. The content of the compounds **D1**, **D2**, and **D3** ranged from 5% to 15%. The mCP having a high triplet energy (ET = −2.91 eV) and a very deep HOMO level serves as a conventional wide gap matrix for fabrication of efficient electrophosphorescent and light emitting diodes with delayed fluorescence (TADF) [48].

**Figure 4:**
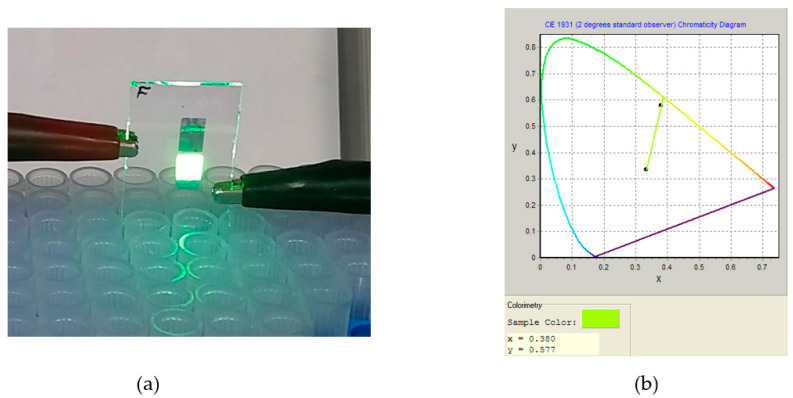
(**a**) OLED based on EML with the **D3** (10 wt %)/mCP composite; (**b**) the CIE chromaticity diagram recorded at 8 V.

**Figure 5:**
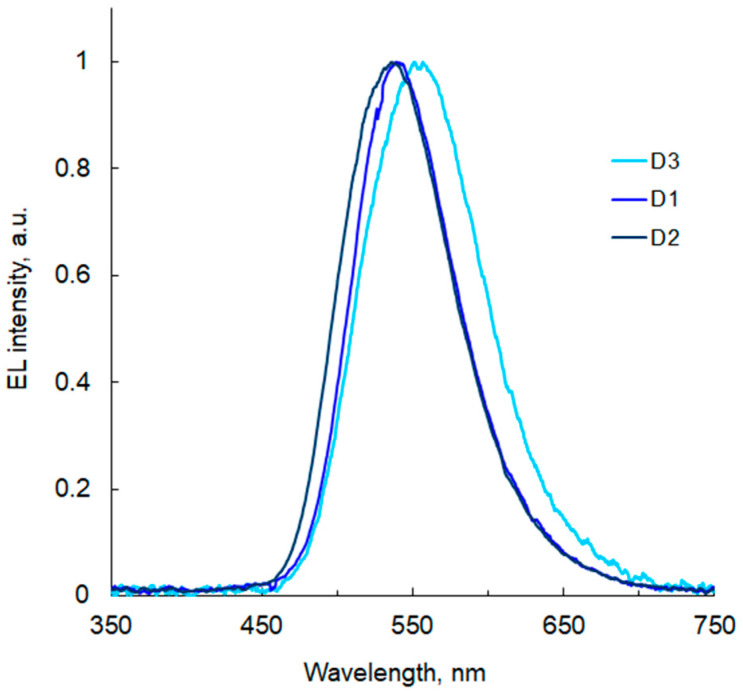
EL spectra of the studied OLEDs.

The best electroluminescent (EL) characteristics were obtained for OLED based on the **D1**–**3** (10 wt %)/mCP composites. The peaks of the EL spectra are in the green-yellow spectral region of 540–560 nm (Table 3, Figure 4) and shift towards the long-wavelength region relative to the PL peaks by 20–40 nm (Figure 5). The position of the maximum EL for **D2** and **D3**, in contrast to **D1**, is noticeably red shifted and depends on the concentration in the mCP matrix. It can be explained both by the different polarizing effect of the matrix on the donor fragments of these compounds (carbazole or triphenylamine) and by the complex behavior of carbazole groups in **D2** and **D3** during the EL process, when different types of excimers and electrometers can be realized, as well as for PVK [57].

**Figure 6:**
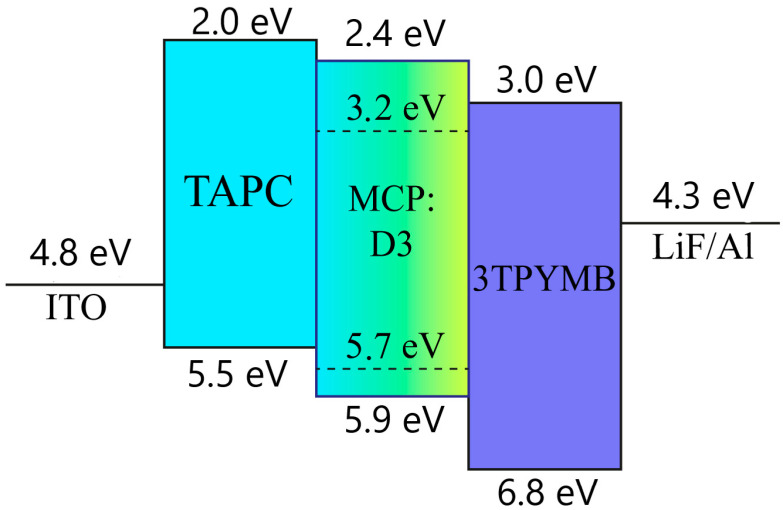
Energy band diagram of OLED structure ITO/TAPC (60 nm)/**D3** (10 wt %): mCP (25 nm)/TPYMB(30 nm)/LiF (1 nm)/Al.

**Figure 7:**
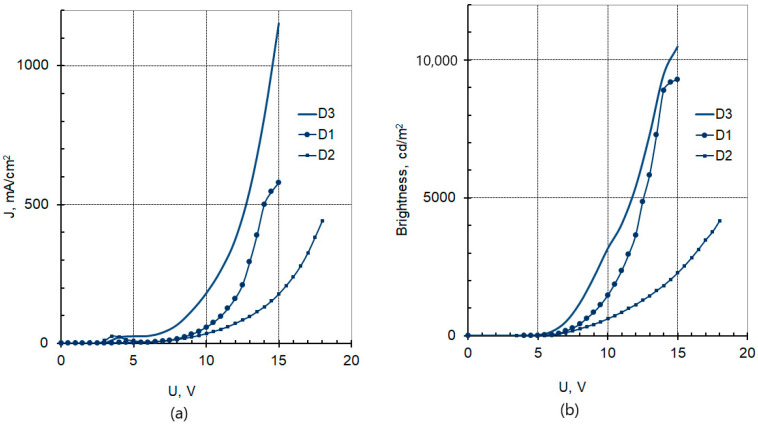
(**a**) Voltage-current density and (**b**) voltage-brightness characteristics of the OLEDs based on EML with the **D1**–**3/**mCP composites.

In OLED with a **D1**–**3**/mCP composite, charge carriers can directly transfer to HOMO and LUMO of **D3** from adjacent hole (TAPC) and electronic (3TPYMB) transport layers, respectively (Figure 6). In this case, electrons can transfer barrier-free into the EML from ETL, and holes from HTL, overcoming the barrier of 0.2 eV. This OLED demonstrated the best characteristics compared to other ones, the maximum brightness at 15V and current density of 1150 mA/cm^2^ reached 10,500 cd/m^2^ (Figure 7a,b), and the maximum current efficiency −2.7 cd/A. A turn-on voltage for this sample is of 4.0 V, which is the lowest value among the studied samples. The brightness of device with **D3** is two times higher than that with **D1**, which in turn is twice then that with **D2** at a voltage of 10 V (Figure 7b), despite the fact that compound **D3** has a lower PL quantum yield compared to **D1** and **D2**. The better performance of the device with **D3** results from the well-matched energy levels as well as from the relatively high and balanced mobility of holes and electrons (Table 2). In spite of the high PL quantum yield, the lowest brightness, the luminous and current efficiency were observed for device using the **D2** compound**.** This can be rationalized by the imbalance of the electrons and holes mobility in the **D2** (10 wt %)/mCP composite layer, with the mobility of electrons is twice higher than the mobility of holes. Hence, the recombination zone in the EML is located at the interface with the HTL.

## 3. Materials and Methods

### 3.1. General Information

All the reactions were carried out under argon atmosphere and the solvents were distilled from appropriate drying agents prior to use. All reagents were used as purchased from Sigma-Aldrich (Munich, Germany). 4-Bromo-7-(4-methoxyphenyl)-2,1,3-benzothiadiazole (**1**) [33], 2,6-dimethyl-4-(4,4,5,5-tetramethyl-1,3,2-dioxaborolan-2-yl)aniline [37] were synthesized according to published procedure, analytical data was in accordance with the literature data. Analytical TLC was performed with Merck silica gel 60 F 254 plates (Darmstadt, Germany); visualization was accomplished with UV light or iodine vapors. Chromatography was carried out using Merck silica gel (Kieselgel 60, 0.063–0.200 mm) and petroleum ether/ethyl acetate as an eluent. The NMR spectra were obtained with Bruker AV-400 (Karlsruhe, Germany) (400 MHz ^1^H, 101 MHz ^13^C, 376 MHz ^19^F) using TMS and CCl_3_F as references for ^1^H and ^19^F NMR spectra respectively.

### 3.2. Preparation and Characterization of Novel Compounds

#### 3.2.1. 9-(5-Bromo-2-methylphenyl)-9H-carbazole (**3**)

Under argon in a Schlenk tube with a magnetic stirring bar, carbazole (618 mg, 3.7 mmol, 0.5 equiv.) and Cs_2_CO_3_ (12 g, 36.8 mmol, 5.0 equiv.) were suspended in 20 mL of dry and degassed DMF. The suspension was stirred for 30 min at ambient temperature before 5-bromo-2-fluoro1,3-dimethylbenzene (1.4, 7.4 mmol, 1.0 equiv.) was added. Then, the reaction mixture was stirred at 150 °C (oil bath temperature) for 24 h. After cooling to room temperature, the mixture was poured into water and extracted with ethyl acetate (3 × 70 mL). The combined organic phases were washed with brine, dried over anhydrous MgSO_4_, filtered, and concentrated under reduced pressure. Purification by chromatography (eluent–hexane:ethyl acetate 20:1) gave the product (847 mg, 68%) as a white solid. M.p. 79–81 °C. ^1^H NMR (400 MHz, Chloroform-d) δ 8.16 (dd, *J* = 7.7, 1.0 Hz, 2H), 7.58 (d, *J* = 8.3 Hz, 1H), 7.54 (s, 1H), 7.41 (t, *J* = 7.7 Hz, 2H), 7.35 (d, *J* = 8.1 Hz, 1H), 7.30 (t, *J* = 7.5 Hz, 2H), 7.05 (d, *J* = 8.2 Hz, 2H), 1.94 (s, 3H). ^13^C NMR (101 MHz, Chloroform-d) δ 141.0, 136.7, 133.0, 132.4, 132.0, 126.2, 123.3, 120.5, 120.0, 119.9, 109.8, 17.4. HRMS: calcd for C_19_H_15_BrN [M + H]^+^: 336.0388; found: 336.0381.

#### 3.2.2. 9-(2-Methyl-5-(4,4,5,5-tetramethyl-1,3,2-dioxaborolan-2-yl)phenyl)-9H-carbazole (**4**) 

Under argon in a Schlenk tube with a magnetic stirring bar, 9-(5-bromo-2-methylphenyl)-9H-carbazole (**3**) (505 mg, 1.5 mmol, 1.0 equiv.), KOAc (441 mg, 4.5 mmol, 3 equiv.), bis(pinacolato)diboron (419 mg, 1.65 mmol, 1.1 equiv.) were added followed by dry dioxane (15 mL). The solution was degassed by argon before adding PdCl_2_(dppf) (22 mg, 0.02 equiv.). Then, the reaction mixture was stirred at 95 °C (oil bath temperature) for 24 h. After cooling to room temperature, the mixture was poured into water and extracted with dichloromethane (3 × 10 mL). The combined organic phases were washed with brine, dried over anhydrous MgSO_4_, filtered, and concentrated under reduced pressure. Purification by chromatography (eluent–hexane:ethyl acetate 15:1) gave the product (403 mg, 70%) as a white solid. M.p. 111–112 °C. ^1^H NMR (400 MHz, CDCl_3_) δ 8.15 (d, *J* = 7.7 Hz, 2H), 7.87 (d, *J* = 7.5 Hz, 1H), 7.79 (s, 1H), 7.48 (d, *J* = 7.6 Hz, 1H), 7.38 (t, *J* = 7.6 Hz, 2H), 7.29–7.24 (m, 2H), 7.03 (d, *J* = 8.1 Hz, 2H), 2.00 (s, 3H), 1.32 (s, 12H); ^13^C NMR (101 MHz, CDCl_3_) δ 141.4, 140.9, 135.9, 135.8, 135.1, 131.1, 125.9, 123.1, 120.4, 119.5, 110.0, 84.1, 25.0, 17.9, missing one carbon (C-B) due to interaction with the boron atom. HRMS: calcd for C_25_H_27_BNO_2_ [M + H]^+^: 384.2134; found: 384.2138.

#### 3.2.3. General Procedure for the Suzuki Synthesis of **2**, **D2** and **D3**

A 25 mL round-bottom flask, equipped with a magnetic stir bar and a reflux condenser, was charged with 4-bromo-7-(4-methoxyphenyl)-2,1,3-benzothiadiazole (**1**) (0.5 mmol), ArBPin or ArB(OH)_2_ (1.2 equiv.), 1,4-dioxane-water mixture (3:1, 8 mL), NaHCO_3_ (3 equiv.) and Pd(PPh_3_)_2_Cl_2_ (5 mol %). The resulting mixture was deaerated with argon and refluxed under argon for 6 h. On completion, the mixture was poured into water and extracted with dichloromethane (3 × 10 mL). The combined organic phases were washed with brine, dried over anhydrous MgSO_4_, filtered, and concentrated under reduced pressure. Purification by chromatography (eluent–hexane:ethyl acetate 30:1) gave analytically pure products.

#### 3.2.4. 4-(7-(4-Methoxyphenyl)benzo[c][1,2,5]thiadiazol-4-yl)-2,6-dimethylaniline (**2**)

Following the general procedure **2** was obtained from **1** and 2,6-Dimethyl-4-(4,4,5,5-tetramethyl-1,3,2-dioxaborolan-2-yl)aniline as red solid (136 mg, 75%). M.p. 227–228 °C. ^1^H NMR (400 MHz, Benzene-*d*_6_) *δ* 8.03 (d, *J* = 8.8 Hz, 2H), 7.80 (s, 2H), 7.53 (d, *J* = 7.4 Hz, 1H), 7.48 (d, *J* = 7.4 Hz, 1H), 7.00 (d, *J* = 8.8 Hz, 2H), 3.37 (s, 3H), 3.09 (br.s, 2H), 2.01 (s, 6H). ^13^C NMR (101 MHz, Benzene-*d*_6_) δ 160.2, 155.0, 143.7, 133.8, 131.8, 131.0, 130.8, 129.9, 127.6, 126.7, 121.4, 114.3, 54.9, 17.8. HRMS: calcd. for C_21_H_20_N_3_OS [M + H]^+^: 362.1327; found: 362.1326.

#### 3.2.5. 4-(7-(4-Methoxyphenyl)benzo[c][1,2,5]thiadiazol-4-yl)-2,6-dimethyl-*N*,*N*-diphenylaniline (**D1**)

Under argon in a Schlenk tube with magnetic stirring bar, **2** (128 mg, 0.35 mmol), bromobenzene (2.2 equiv.), palladium acetate (5 mol %), tri-*tert*-butylphosphonium tetrafluoroborate (10 mol %) and sodium *tert*-butoxide (2.4 equiv.) were dissolved in dry 1,4-dioxane (4 mL). The solution was degassed by argon. Then, the reaction mixture was stirred at 100 °C (oil bath temperature) for 24 h. After cooling to room temperature, the mixture was poured into water and extracted with dichloromethane (3 × 10 mL). The combined organic phases were washed with brine, dried over MgSO4, filtered, and concentrated under reduced pressure. Purification by chromatography (eluent–hexane:ethyl acetate 30:1) gave **D1** as a yellow solid (65 mg, 54%). M.p. 201–203 °C. ^1^H NMR (400 MHz, Benzene-*d*_6_) δ 8.02 (d, *J* = 8.7 Hz, 2H), 7.89 (s, 2H), 7.54 (d, *J* = 7.3 Hz, 1H), 7.47 (d, *J* = 7.4 Hz, 1H), 7.11–7.05 (m, 8H), 7.01 (d, *J* = 8.5 Hz, 2H), 6.82 (t, *J* = 6.5 Hz, 2H), 3.37 (s, 3H), 2.19 (s, 6H). ^13^C NMR (101 MHz, Benzene-*d*_6_) *δ* 160.5, 154.7, 146.5, 143.4, 138.3, 136.9, 133.3, 132.5, 131.1, 130.8, 130.4, 129.6, 127.2, 121.4, 120.3, 114.4, 54.9, 19.3. HRMS: calcd for C_33_H_27_N_3_OS [M]^+^: 513.1875; found: 513.1873.

#### 3.2.6. 4-(3-(9H-Carbazol-9-yl)-4-methylphenyl)-7-(4-methoxyphenyl)benzo[c][1,2,5]thiadiazole (**D2**)

Following the general procedure **D2** was obtained from **1** and 9-(2-methyl-5-(4,4,5,5-tetramethyl-1,3,2-dioxaborolan-2-yl)phenyl)-9H-carbazole as a yellow solid (204 mg, 82%). M.p. 210–212 °C. ^1^H NMR (400 MHz, CDCl_3_) *δ* 8.21 (d, *J* = 7.8 Hz, 2H), 8.15 (d, *J* = 8.0 Hz, 1H), 8.01 (s, 1H), 7.92 (d, *J* = 8.4 Hz, 2H), 7.78 (d, *J* = 7.4 Hz, 1H), 7.67 (dd, *J* = 15.4, 7.7 Hz, 2H), 7.44 (t, *J* = 7.7 Hz, 2H), 7.32 (t, *J* = 7.5 Hz, 2H), 7.23 (d, *J* = 8.2 Hz, 2H), 7.08 (d, *J* = 8.5 Hz, 2H), 3.90 (s, 3H), 2.10 (s, 3H). ^13^C NMR (101 MHz, CDCl_3_) *δ* 160.0, 154.3, 154.0, 141.2, 137.4, 136.8, 136.4, 133.3, 131.9, 131.3, 130.6, 129.9, 129.5, 128.1, 127.3, 126.1, 123.2, 120.5, 119.8, 114.2, 110.1, 55.5, 17.7. HRMS: calcd for C_32_H_24_N_3_OS [M + H]^+^: 498.1640; found: 498.1634.

#### 3.2.7. 4-(4-(9H-Carbazol-9-yl)phenyl)-7-(4-methoxyphenyl)benzo[c][1,2,5]thiadiazole (**D3**)

Following the general procedure **D3** was obtained from **1** and 4-(9*H*-carbazol-9-yl)phenylboronic acid as a yellow solid (186 mg, 76%). M.p. 234–236 °C. ^1^H NMR (400 MHz, CDCl_3_) *δ* 8.22 (d, *J* = 8.4 Hz, 2H), 8.19 (d, *J* = 7.8 Hz, 2H), 7.98 (d, *J* = 8.7 Hz, 2H), 7.87 (d, *J* = 7.3 Hz, 1H), 7.78 (dd, *J* = 13.1, 7.9 Hz, 3H), 7.58 (d, *J* = 8.2 Hz, 2H), 7.46 (t, *J* = 7.6 Hz, 2H), 7.33 (t, *J* = 7.4 Hz, 2H), 7.12 (d, *J* = 8.7 Hz, 2H), 3.92 (s, 3H). ^13^C NMR (101 MHz, CDCl_3_) *δ* 160.1, 154.3, 154.1, 140.8, 137.8, 136.6, 133.5, 131.6, 130.7, 130.6, 129.9, 128.5, 127.4, 127.2, 126.1, 123.6, 120.5, 120.2, 114.3, 110.1, 55.6. HRMS (APPI): calcd. for C_31_H_21_N_3_OS [M]^+^: 483.1400; found: 483.1402.

### 3.3. Absorption and Emission Spectra, Photoluminescence Quantum Yield

UV–VIS spectra were measured using an AvaSpec-2048 spectrophotometer (Avantes, The Netherlands). The photoluminescence spectra were recorded at 77 K and 298 K, on the Fluorolog-3 spectrofluorometer system (HORIBA Jobin Yvon S.A.S., France). The excitation source was a 450 W Xenon lamp with Czerny-Turner double monochromators, the registration channel was a R928 photomultiplier with Czerny-Turner double monochromators). Pulsed xenon lamp (150 W) was used for phosphorescence spectra and phosphorescence decay curve measurements. The phosphorescence decay curves were analyzed using the FluoroEssence^TM^ software for calculation of the phosphorescence lifetime values. Quantum yields of solid films photoluminescence and solution photoluminescence were measured by the absolute method. Light of photoluminescence was collected by Quanta-*φ* F-3029-sphere linked with Fluorolog 3 by Fiber-Optics adaptor FL-3000 produced by Horiba Jobin Yvon S.A.S. (France) Photoluminescence quantum yields were calculated by FluoroEssence^TM^ software.

### 3.4. Photoluminescence Decay Measurements

For time-resolved photoluminescence spectroscopy was used an experimental setup based on Q-switch YAG/Nd laser (self-made, Moscow, Russia), high-resolution monochromator (M266 Solar LS, Belarus), a fast PMT H10720 (Hamamatsu, Japan) and an oscilloscope (Tektronix DPO 3054 (USA)). The third harmonic of YAG/Nd laser with wavelength 355 nm, pulse duration 6 ns and repetition rate 10 Hz was used for samples excitation. The photoluminescence decay was measured at a wavelength of 550 nm for all samples.

### 3.5. Voltammetry Studies

The HOMO/LUMO levels were determined by cyclic voltammetry (CV) using an potentiostat Autolab AUT204 (Utrecht, The Netherlands). The CV experiment was carried out at the scan rate of 20 mV/s in a three-electrode, three-compartment electrochemical cell in the glove box with dry argon atmosphere. Platinum sheets served as working and counter electrodes. A 0.2 M solution of tetrabutylammonium hexafluorophosphate (NBu_4_PF_6_, Fluka) in acetonitrile (ACN) was used as an electrolyte. An Ag wire immersed into the electrolyte solution with the addition of 0.1 M AgNO_3_ was used as a pseudo reference electrode (Ag/Ag^+^). It was calibrated against ferrocene/ferricenium couple (−0.039 V vs. Ag/Ag^+^) and its potential was recalculated to the energy scale using −4.988 eV value for Fc/Fc^+^ in ACN. Thus, the energy level of Ag/Ag^+^ in our case is −4.95 eV. The substances investigated (0.2 mg) were dissolved in 0.2 M solution of NBu_4_PF_6_ in ACN and placed into the working compartment of the electrochemical cell. The values of potentials corresponding to the HOMO and LUMO levels were determined by applying a tangent to the onset of anodic and cathodic currents. Obtained voltammograms are presented in the Supplementary Materials.

### 3.6. Charge Mobility Measurements

In thin layers of the composites, charge carrier mobility (*μ*) was measured by using the technique of charge extraction by linearly increasing voltage (CELIV) with metal-insulator-semiconductor (MIS) diode structures. A layer of the **D1**–**3**/mCP (10 wt %) composite of the thickness *d*s ranged between 100 nm and 130 nm was deposited onto SiO_2_/ITO/glass substrate by thermal co-evaporation of mCP and **D1**–**3** in a chamber under 10^−6^ mbar vacuum. Then a 80 nm thick Al top electrode was deposited onto the composite layer also by thermal vacuum evaporation (TVE) of the material under 10^−6^ mbar residual pressure at rate of 1.0 Å/s. A SiO_2_ layer of the thickness *d_i_* = 70 nm was preliminary deposited onto ITO-coated glass by magnetron scattering at 10^−3^ mbar; the layer served as a charge carrier blocking layer. As a result, for measuring the mobility of charge carriers by the MIS-CELIV technique, the samples of the glass/ITO/SiO_2_ (70 nm)/**D1**–**3**/mCP (10%)/Al (80 nm) architecture were prepared. More experimental details and calculations of the charge carrier mobility are described in [54,58,59,60] and Supplementary Materials.

### 3.7. OLED Device Fabrication and Characterization

Organic light-emitting diodes (OLEDs) were fabricated on glass substrates coated with a transparent conductive layer of indium-tin oxides In_2_O_3_:SnO_2_ (ITO). The substrates were pre-cleaned sequentially in an aqueous solution of a detergent, deionized water, and isopropyl alcohol in an ultrasonic bath for 10 min, respectively. Then, the substrates were dried in an Ar flow and treated in oxygen plasma for 2.5 min. The cleaned substrates were placed in a vacuum chamber in a special holder. Then the process of sequential deposition of hole transport, light-emitting, electron transport layers was carried out using TVE at a residual pressure of 4 × 10^−6^ mbar. After that, the samples were placed in special masks, through which a LiF (1 nm)/Al (80 nm) cathode was applied. The EL spectra of OLEDs were recorded on an AvaSpec-2048 spectrophotometer (Avantes, The Netherlands). Voltage–current and voltage–brightness characteristics were measured with Keithley 2601 Source-Meter (USA), Keithley 6485 pico-ammeter and TKA-04/3 luxmeter-brightness meter (St.-Petersburg, Russia). The thicknesses of the films were determined using MII-4 interferometer (LOMO, St.-Petersburg, Russia). The preparation of OLED samples and measurements of their spectral and optoelectronic characteristics were performed at room temperature under argon atmosphere.

### 3.8. DFT Calculations

Geometry optimizations were performed without constraints at the B3LYP/6-31G(d) level using Gaussian 09 software (revision D.01) with corrections for solvation in xylene-mixture (the PCM model). The optimized geometry was verified to have no negative frequencies. Then TD-DFT was adopted at the same level to estimate the excitation energies (*E*(*S*_1_), *E*(*T*_1_), and *E*(*T*_2_)) based on the vertical excitations. The first five singlet and triplet excited states of were considered. The UV-VIS spectra were calculated using the range-separated CAM-B3LYP functional, which has been shown to better predict UV-VIS spectra of organic dyes [61]

## 4. Conclusions

In summary, an efficient synthetic procedure for the preparation of three new luminophores with different configuration of aryl linker has been elaborated via combination of Pd-catalyzed Suzuki and double Buchwald–Hartwig cross-coupling reactions. All compounds obtained have a sufficiently high quantum yields in film (in mCP matrix). Despite the presence of a charge-transfer state and small values of ∆E_ST_, the TADF effect for these compounds was not detected under studied conditions. It was revealed that the mCP composites based on compounds **D2** and **D3** with benzothiadiazole and carbazole units demonstrate enhanced electrons and holes mobility reaching the best magnitudes in the case of **D3**. Compound **D1** containing diphenylamine fragment does not significantly affect the hole mobility in the composite. The highest brightness, current and luminous efficiency were observed for OLED with **D3** in the light-emitting layer, which does not have the highest photoluminescence quantum yield among the studied compounds. We believe that it is precisely the balanced mobility of electrons and holes along with their high values play a key role in the results obtained for the **D3**/mCP composite. This combination provides efficient recombination of electrons and holes with a simultaneous high rate of their injection into the light-emitting layer. Therefore, the studied compounds **D1**, **D2**, and **D3** can serve both as light emitters with a high quantum yield of photoluminescence and as well as active transport centers for charge carriers in the developed OLEDs.

## Figures and Tables

**Table 1:** Absorption and emission characteristics of **3a**–**c** at 298 and 77 K ^a^.

Substance	λ^abs^_max_ 298 K, nm ^b^	λ^PL^_max_ 298 K, nm ^b^	λ^PL^_max_ 77 K, nm ^b^	λ^P^_max_ 77 K, nm ^b^	λ^PL^_max_ 298 K, nm ^c^	φ^PL^ 298 K, % ^c^
**D1**	420	536	508	520	539	98.6
**D2**	404	506	482	488	523	91.1
**D3**	412	517	498	502	534	49.2

^a^ Absorption (λ^abs^_max_) and emission (λ^PL^_max_—for photoluminescence; λ^P^_max_—for phosphorescence) maxima, photoluminescence total quantum yield in integrating sphere (φ^PL^). λ excitation for luminescence—375–400 nm, delay for phosphorescence—1 ms. ^b^ Measured in m-xylene, 10^−5^ M, optical length 1 cm. ^c^ Measured in film (in mCP matrix, 10 wt %).

**Table 2:** LUMO-HOMO levels (eV) and charge carrier mobility (cm^2^V^−1^s^−1^).

Material	LUMO	Electron Mobility	HOMO	Hole Mobility
mCP	−2.400	3.37·10^−4^	−5.900	3.89·10^−4^
**D1**	−3.452	3.86·10^−4^	−5.238	2.49·10^−4^
**D2**	−3.442	1.88·10^−3^	−5.968	8.73·10^−4^
**D3**	−3.263	2.68·10^−3^	−5.785	3.97·10^−3^

**Table 3:** EL characteristics of the studied OLEDs with different light-emitting layers.

Ligt-Emitting Layer	U_on_, V	Brightness cd/m^2^ U = 15V	Max. Efficiency		CIE		λ_max_ EL, nm
			Current cd/A	Luminous lm/W	X	y	
**D1**/mCP	4.4	9300	2.04	1.09	0.354	0.580	542
**D2**/mCP	4.6	2270	1.80	0.75	0.330	0.578	539
**D3**/mCP	4.0	10,500	2.70	0.91	0.380	0.577	558

## Data Availability

Data is contained within the article and Supplementary Materials.

## Supplementary Materials

The following are available online: copies of ^1^H and ^13^C NMR spectra for all new compounds, cyclic voltammograms for **D1**–**3**, and diagrams for MIS-CELIV experiment [62]; Table S1: selected molecular orbitals of **D1**–**3** at the B3LYP/6-31G(d) level (isovalue = 0.02 a.u.). Energies of the orbitals are given in parentheses in eV. Table S2: geometry of the ground state and energy parameters of excitated states of **D1**–**3** as predicted from DFT (at the B3LYP/6-31G(d) level using the PCM solvation model with xylene-mixture as a solvent). Table S3: general analysis of the absorption bands of **D1**–**3** by TD DFT calculations at the CAM-B3LYP/6-31G(d) level.

## Sample Availability

Samples of all of the compounds are available from the authors.
