# Supplementary material for: New Unsymmetrically Substituted Benzothiadiazole-Based Luminophores: Synthesis, Optical, Electrochemical Studies, Charge Transport, and Electroluminescent Characteristics
Source: Molecules. 2021 Dec 15;26(24):7596. doi: 10.3390/molecules26247596 (PMC8705470; doi:10.3390/molecules26247596)
Supplement: Supplementary file 1 [file molecules-26-07596-s001.zip › molecules-1508480-supplementary.pdf]

Supporting information *for*

Novel unsymmetrically substituted benzothiadiazole-based luminophores: synthesis, optical, electrochemical studies, charge transport and electroluminescent characteristics.

Pavel S.Gribanov, Dmitry A. Loginov, Dmitry A.Lypenko, Artem V.Dmitriev, Sergey I.Pozin, Alexey E. Aleksandrov, Alexey R. Tameev, Igor L. Martynov, Andrey Yu. Chernyadyev, Sergey N.Osipov

Table of contents

Voltammetry studies....................1
Charge mobility measurements....................3
References....................5
DFT calculations....................6
NMR spectra....................8

Voltammetry studies

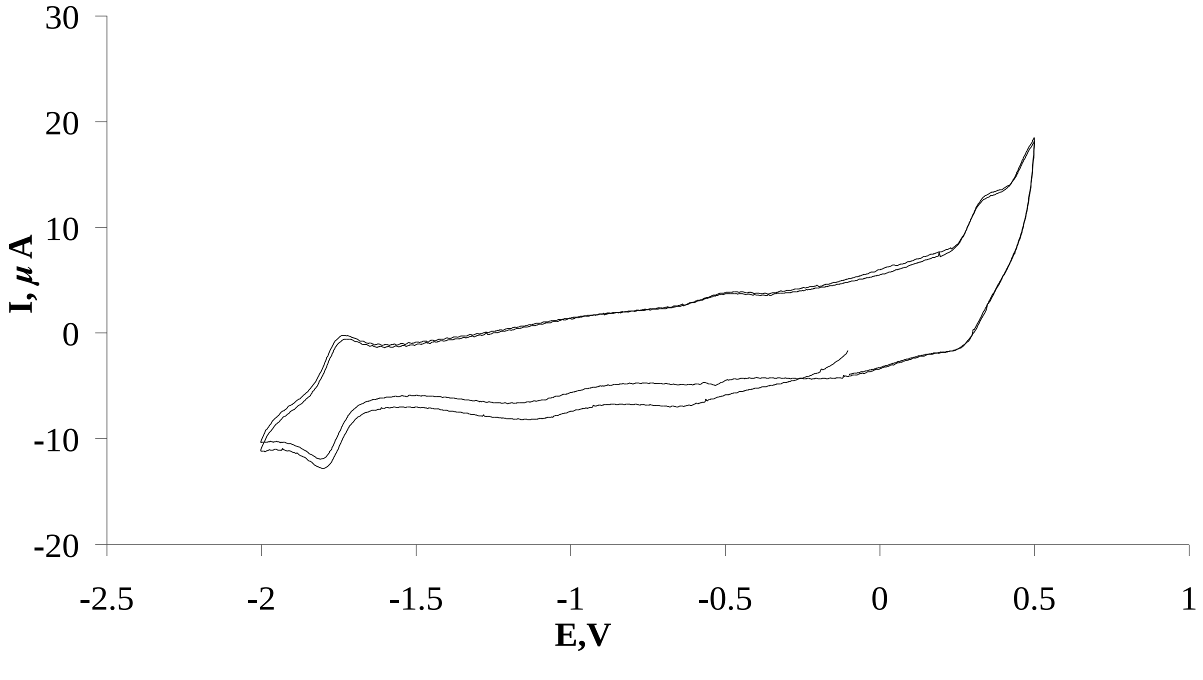

Figure S1. Cyclic voltammogram of **D1**.

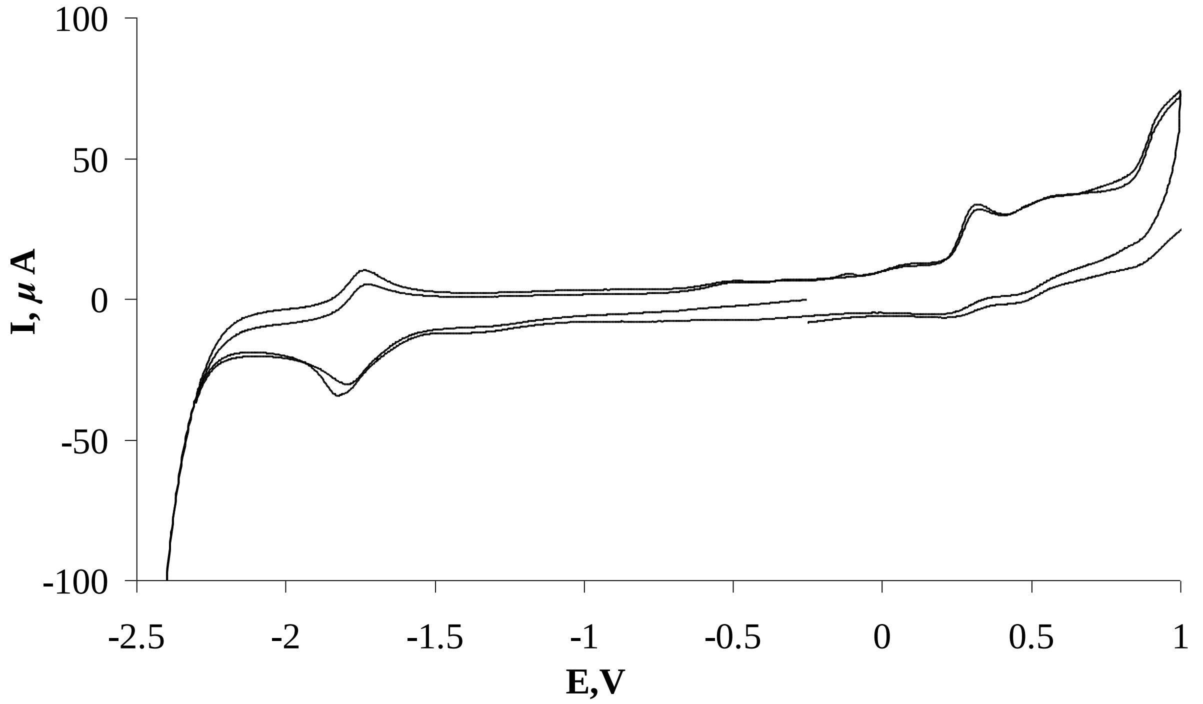

Figure S2. Cyclic voltammogram of **D2**.

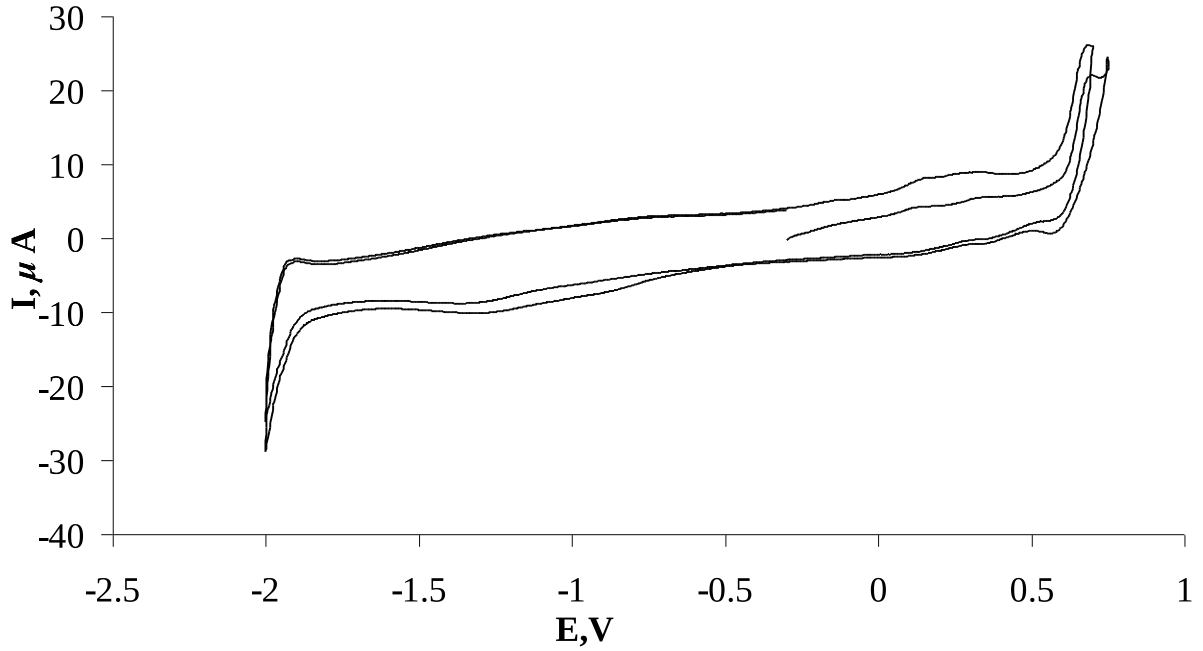

Figure S3. Cyclic voltammogram of **D3**.

Charge mobility measurements

In thin layers of the composites, charge carrier mobility (μ) was measured by using the technique of charge extraction by linearly increasing voltage (CELIV) with metal-insulator-semiconductor (MIS) diode structures. A thin layer of the mCP/PSG (10 wt.%) composite of the thickness d_s ranged between 100 nm and 130 nm was deposited onto the SiO_2/ITO/glass substrate by co-deposition in vaccuum chamber. Then a 80 nm thick Al top electrode was deposited onto the composite layer by thermal evaporation of the material under 10^{-6} mbar vacuum at rate of 1 Å/s. A charge carrier blocking SiO_2 layer of the thickness d_i = 70 nm was preliminary deposited onto ITO-coated glass by magnetron scattering at 10^{-3} mbar.

The CELIV set-up included a digital USB-oscilloscope (DL-Analog Discovery, Digilent Co.), which played roles of master pulse generator and transient current pulse monitor. *RC* constants were at least a factor of 20 smaller than the time scales of interest. The bias was swept in the range between 10 and 100 kHz.

A small charge extraction regime of the MIS-CELIV experiment was used. The condition $\Delta j \leq j(0)$ where Δj is a maximum current of the unipolar charge carriers and j(0) is a capacitance current [1]. The corresponding small-charge transit time t_{max} for the sheet of carriers to reach the extracting contact defines the mobility as following [1]:

$$\mu = \frac{2d_s^2}{At_{max}^2(1+f)}$$

where the ratio between the geometric capacitances of the organic semiconductor and the SiO_2 insulator layers $f \equiv (\varepsilon_s\ d_i)/(\varepsilon_i\ d_s)$ is ranged between ~0.34 and ~0.45 for dielectric constant is ε_i =3.9 and ε_s=~2.5 for SiO_2 and mCP/PSG (10 wt.%) composite, respectively.

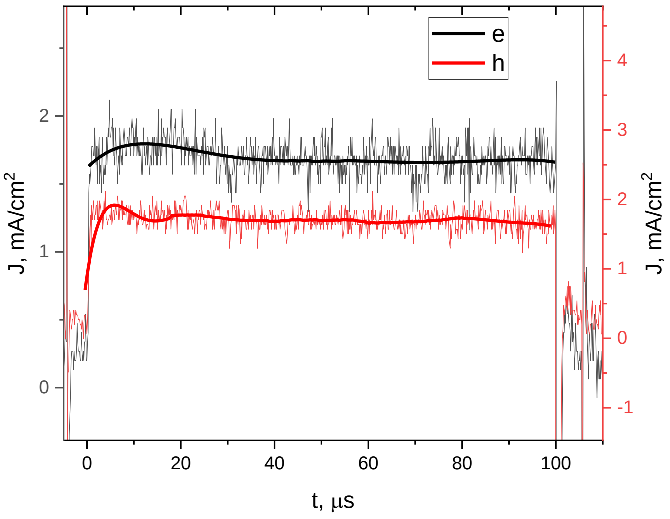

Figure S4. MIS-CELIV transient current of holes and electrons in mCP

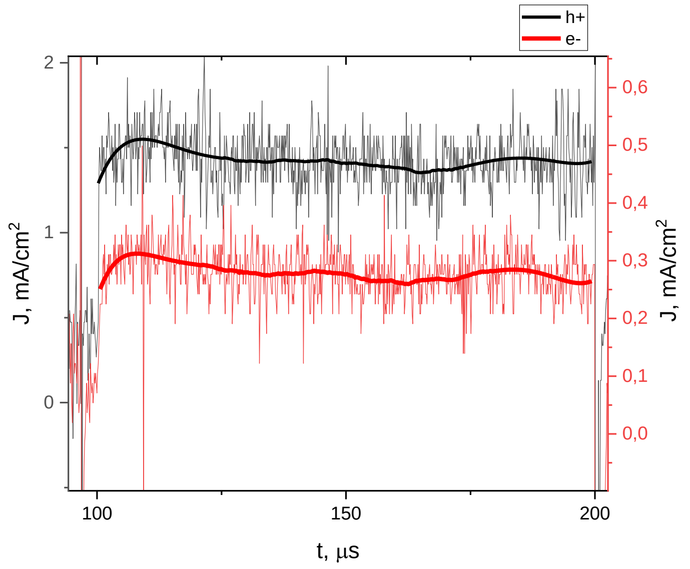

Figure S5. MIS-CELIV transient current of holes and electrons in **D1**/mCP composite

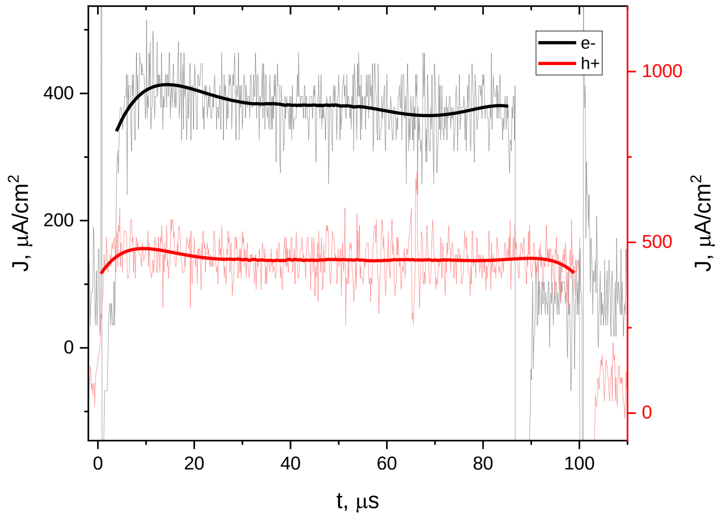

Figure S6 . MIS-CELIV transient current of holes and electrons in **D2/**mCP composite

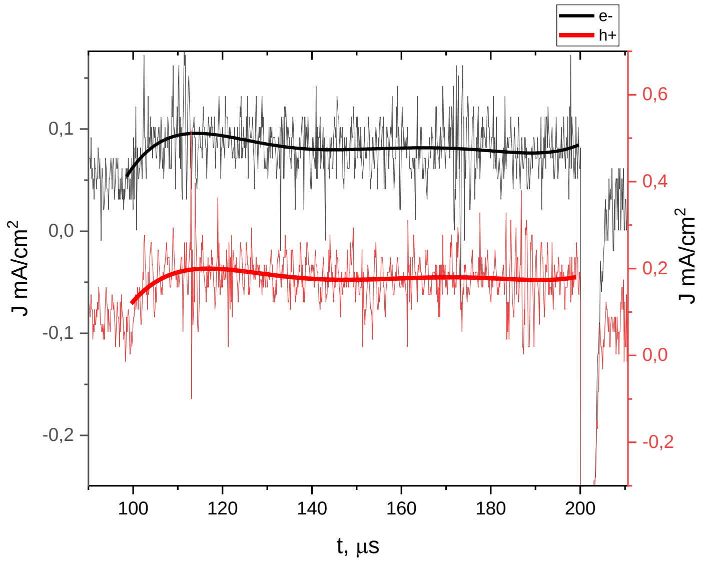

Figure S7. MIS-CELIV transient current of holes and electrons in **D3**/mCP composite

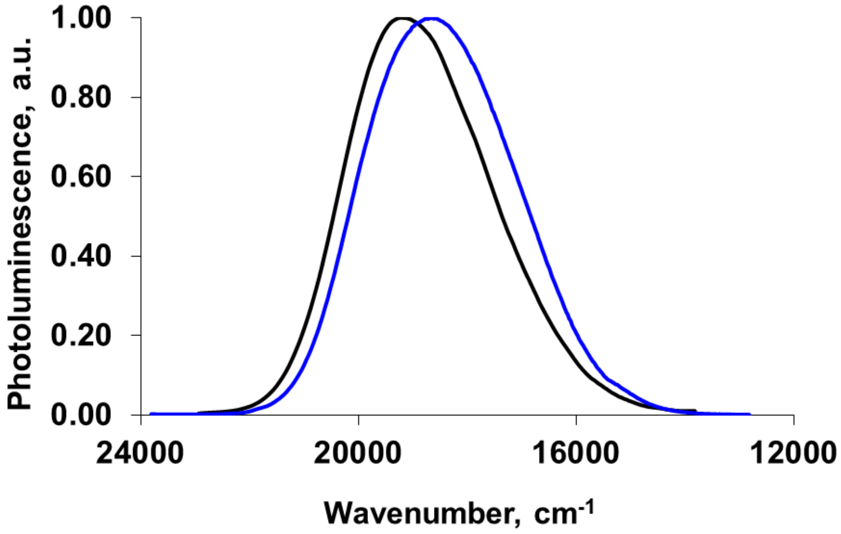

Figure S8. Solvatochromism for **D3**. Black – PL in *m*-xylene, blue – PL in chlorobenzene. Concentration 10^{-5} M, λ_{ex} = 375-400 nm.

References

[1] Sandberg, O.J.; Nyman, M.; Dahlstrom, S.; Sanden, S.; Torngren, B.; Smatt, J.-H.; Osterbacka, R. On the validity of MIS-CELIV for mobility determination in organic thin-film devices. *Appl. Phys. Lett*. **2017**, *110*, 153504.

DFT calculations

Table S1. Selected molecular orbitals of **D1-3** at the B3LYP/6-31G(d) level (isovalue = 0.02 a.u.). Energies of the orbitals are given in parentheses in eV.

Compound	HOMO−1	HOMO	LUMO
**D1**	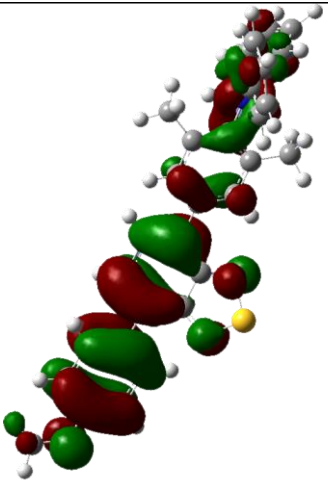 −5.58	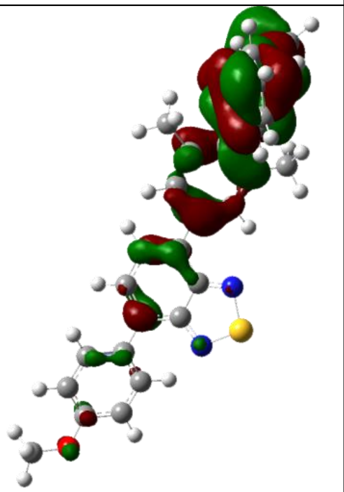 −5.05	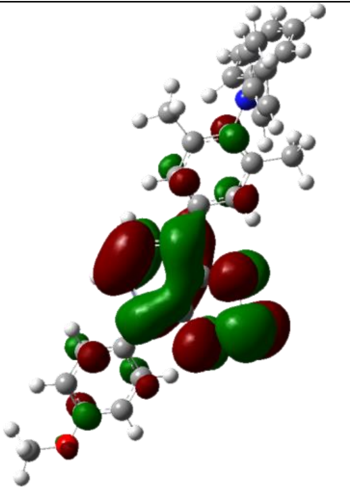 −2.36
**D2**	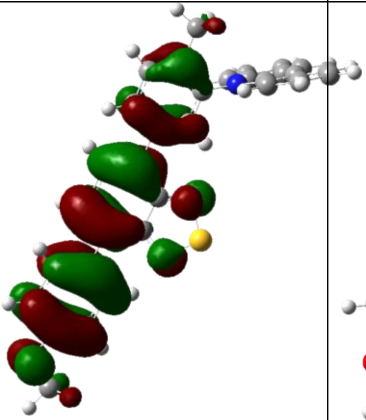 −5.56	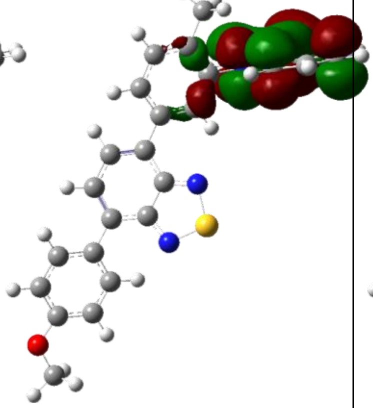 −5.41	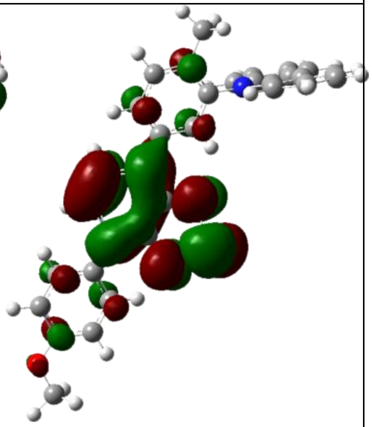 −2.42
**D3**	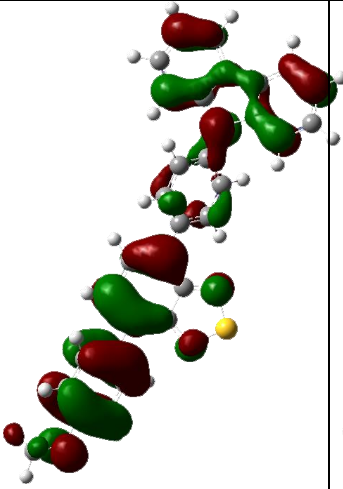 −5.69	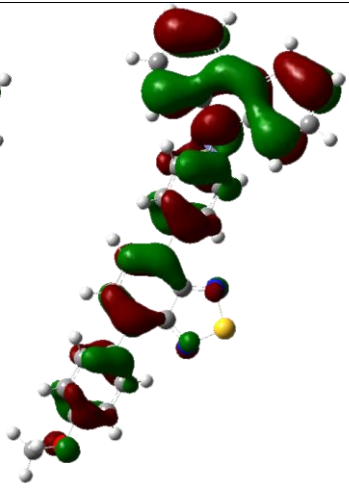 −5.33	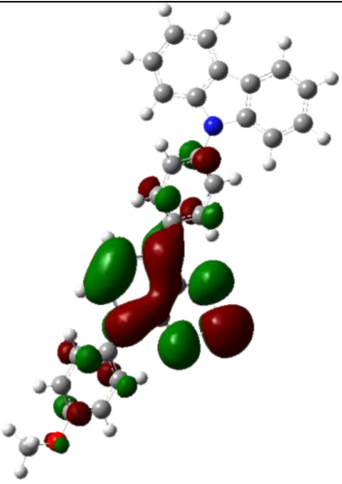 −2.44

Table S2. Geometry of the ground state and energy parameters of excitated states of **D1-3** as predicted from DFT (at the B3LYP/6-31G(d) level using the PCM solvation model with xylene-mixture as a solvent).

Compound	θ_1,[a] °	θ_2,[b] °	θ_3,[c] °	$E(S_1)$,[d] eV	$E(T_1)$,[d] eV	$E(T_2)$,[d] eV
**D1**	-	36.9	36.5	2.289	1.765	2.377
**D2**	78.5	36.1	35.0	2.583	1.800	2.581
**D3**	54.3	36.8	36.1	2.478	1.783	2.604

[a] θ_1 is the dihedral angle between carbazole and aryl linker. [b] θ_2 is the dihedral angle between aryl linker and benzothiadiazole. [c] θ_3 is the dihedral angle between benzothiadiazole and 4-MeO-phenyl substituent. [d] $E(S_1)$ is the energy of the first excited singlet state. $E(T_1)$ and $E(T_2)$ are the energy levels of the first and second excited triplet states.

Table S3. General analysis of the absorption bands of **D1-3** by TD DFT calculations at the CAM-B3LYP/6-31G(d) level.

Compound	Experimental absorption maxima (nm)	Calculated absorption maxima (nm)	Calculated oscillator strengths	Main MOs responsible for excitation[a]
**D1**	420	395	0.6218	HOMO → LUMO (43%),<br>HOMO–1 → LUMO (52%),<br>HOMO–2 → LUMO (2%)
**D2**	404	389	0.5594	HOMO–1 → LUMO (93%)
**D3**	412	392	0.6548	HOMO → LUMO (55%),<br>HOMO–1 → LUMO (39%),<br>HOMO–3 → LUMO (3%)

[a] Values in parentheses give the percentage contribution of the corresponding orbitals to the total transition.

NMR spectra

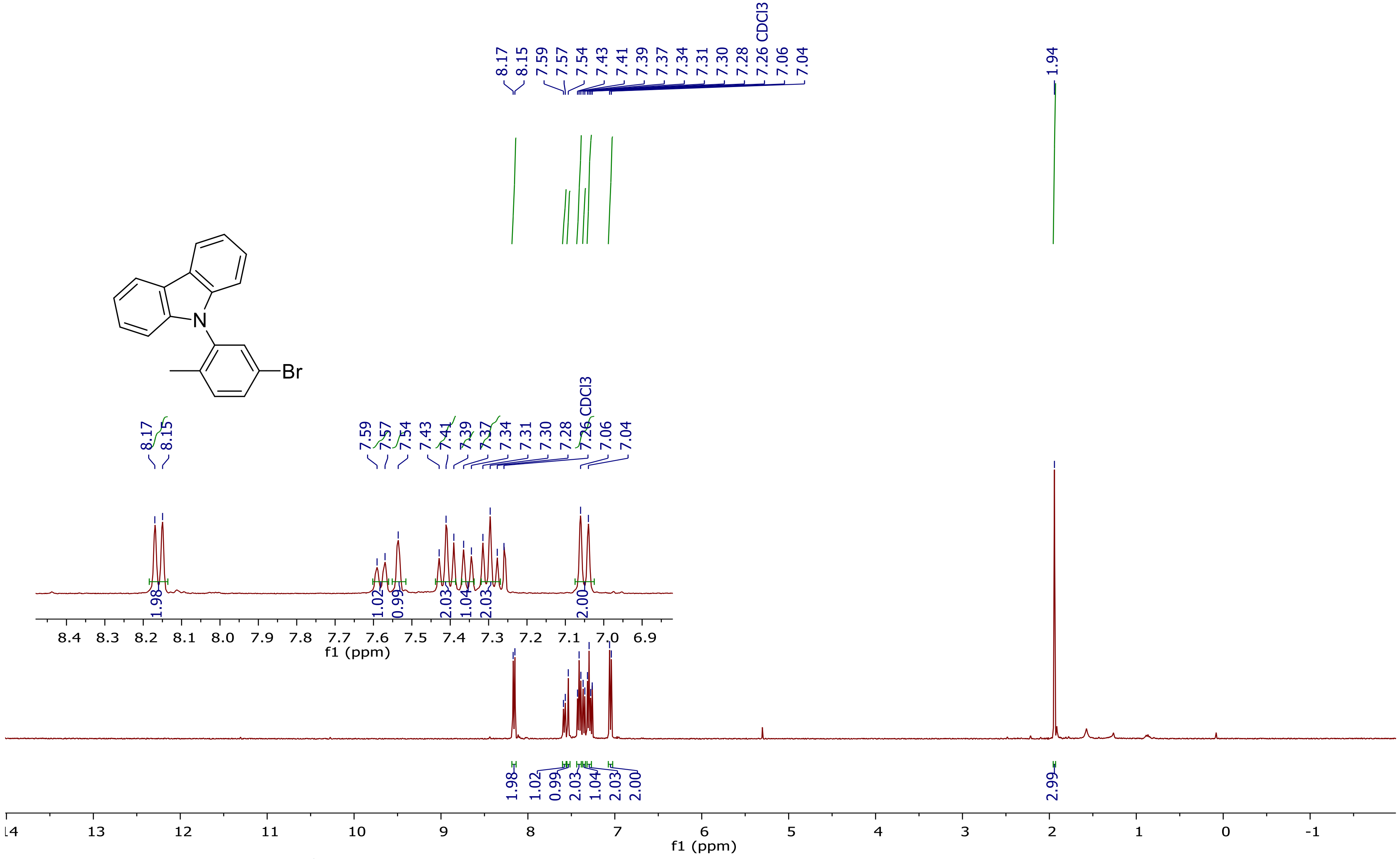

Figure S9. ^{1}H (400 MHz, Chloroform-*d*) of NMR 9-(5-bromo-2-methylphenyl)-9*H*-carbazole (3)

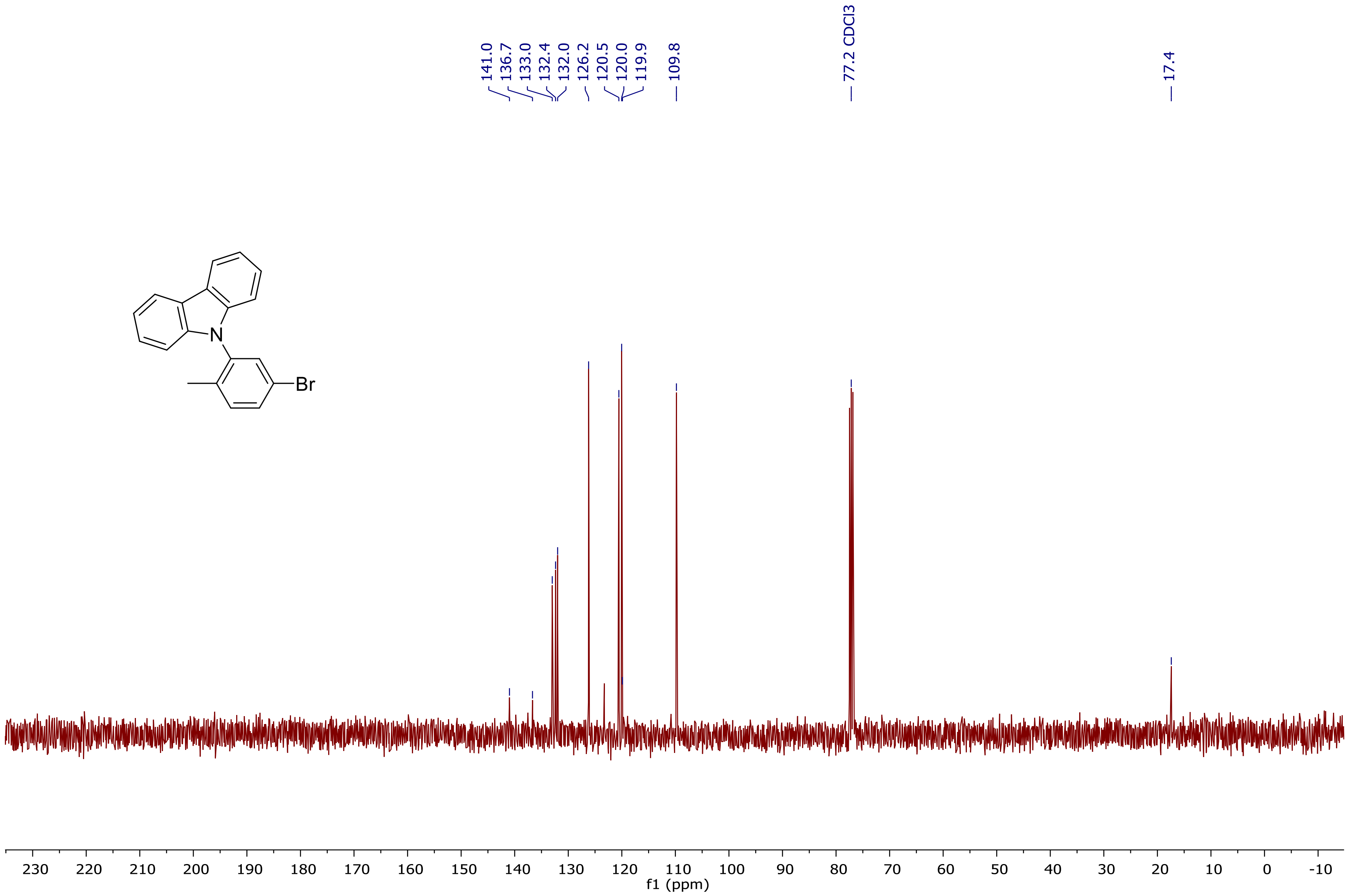

Figure S10. $^{13}C\{^1H\}$ **NMR (101 MHz, Chloroform-*d*) of 9-(5-bromo-2-methylphenyl)-9*H*-carbazole (3)**

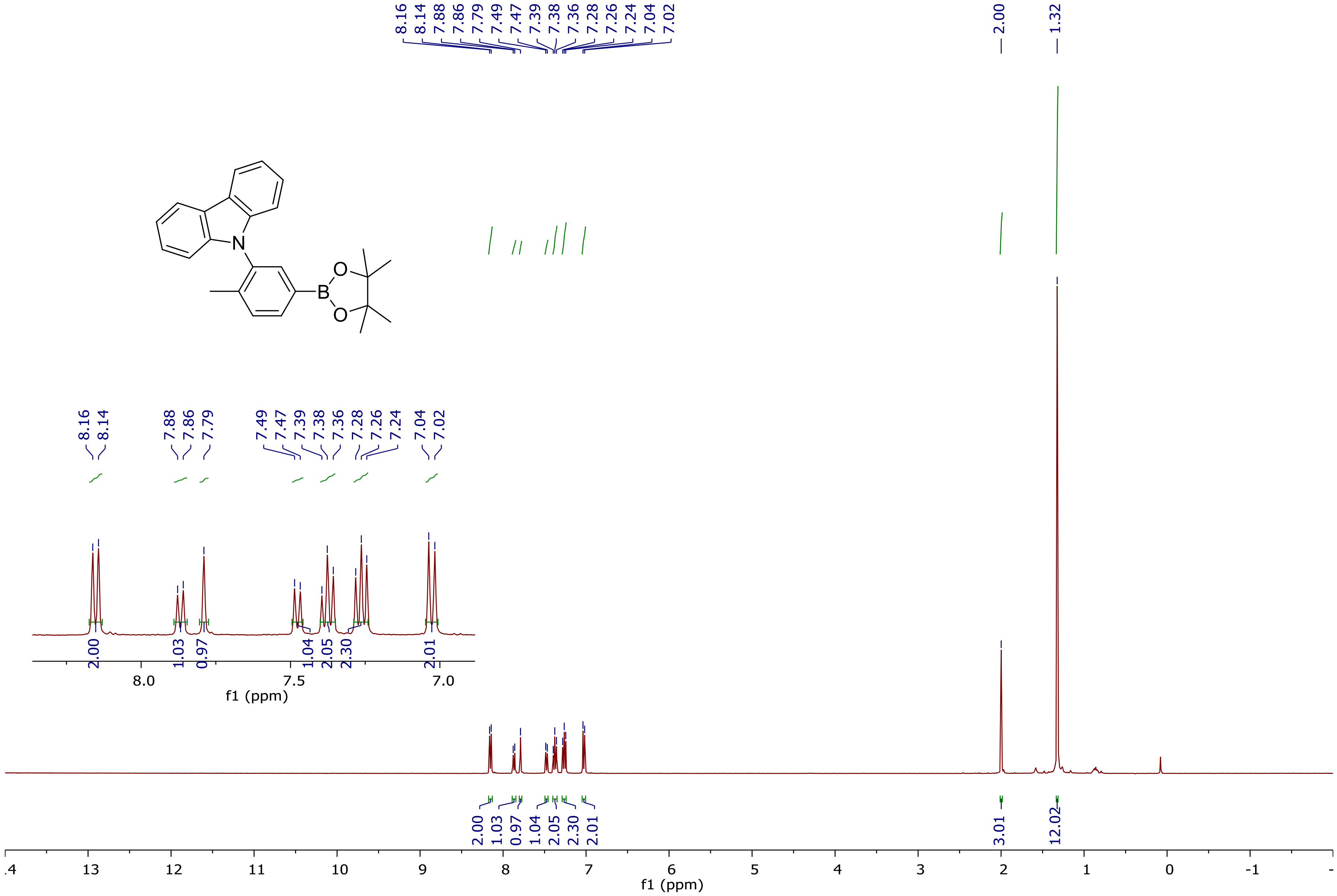

Figure S11. **^{1}H (400 MHz, Chloroform-*d*) of 9-(2-methyl-5-(4,4,5,5-tetramethyl-1,3,2-dioxaborolan-2-yl)phenyl)-9*H*-carbazole (4)**

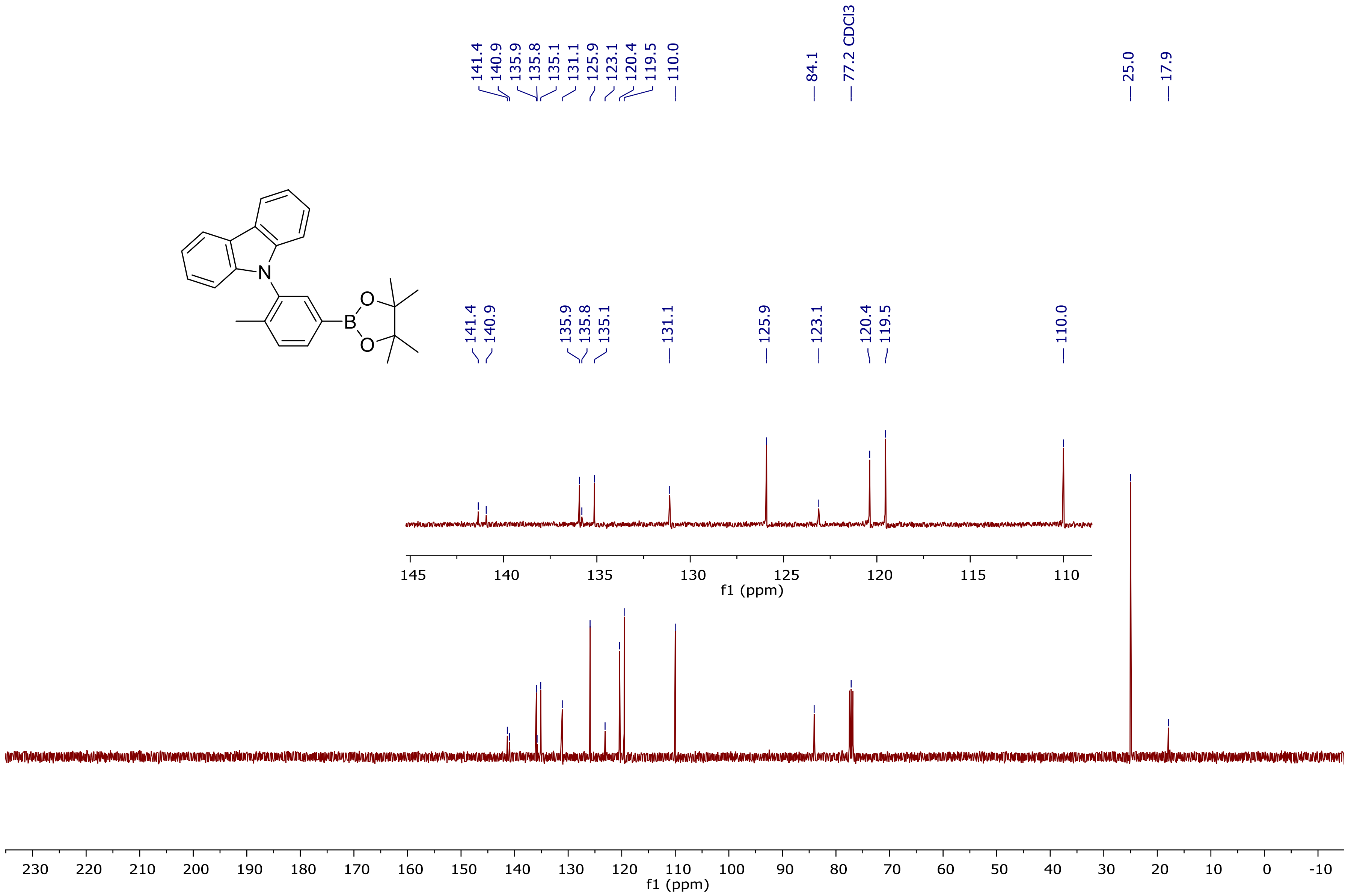

Figure S12. $^{13}C\{^{1}H\}$ **NMR (101 MHz, Chloroform-*d*) of 9-(2-methyl-5-(4,4,5,5-tetramethyl-1,3,2-dioxaborolan-2-yl)phenyl)-9*H*-carbazole (4)**

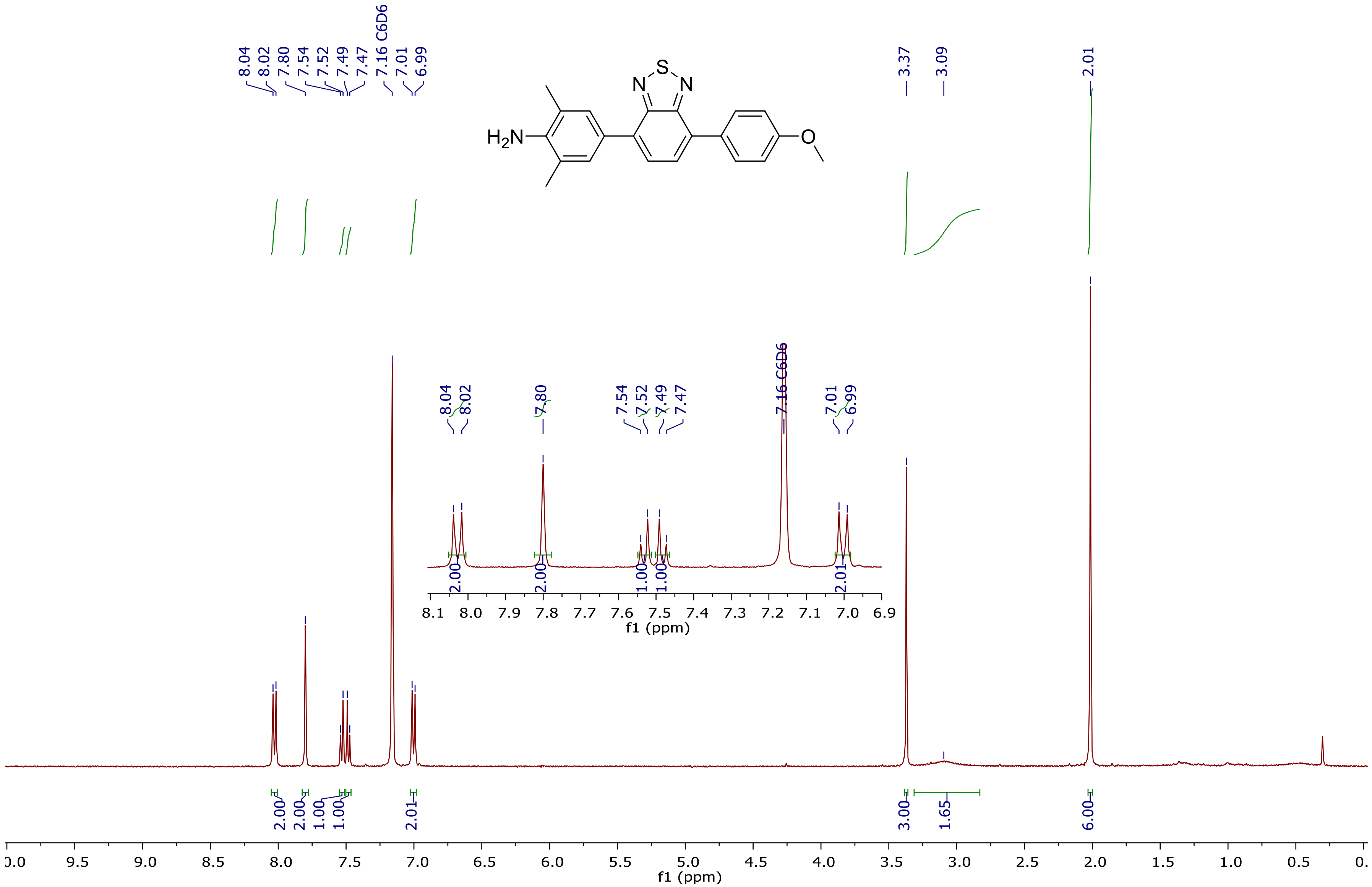

Figure S13. ^{1}H NMR (400 MHz, Benzene-*d*6) of 4-(7-(4-methoxyphenyl)benzo[c][1,2,5]thiadiazol-4-yl)-2,6-dimethylaniline (2)

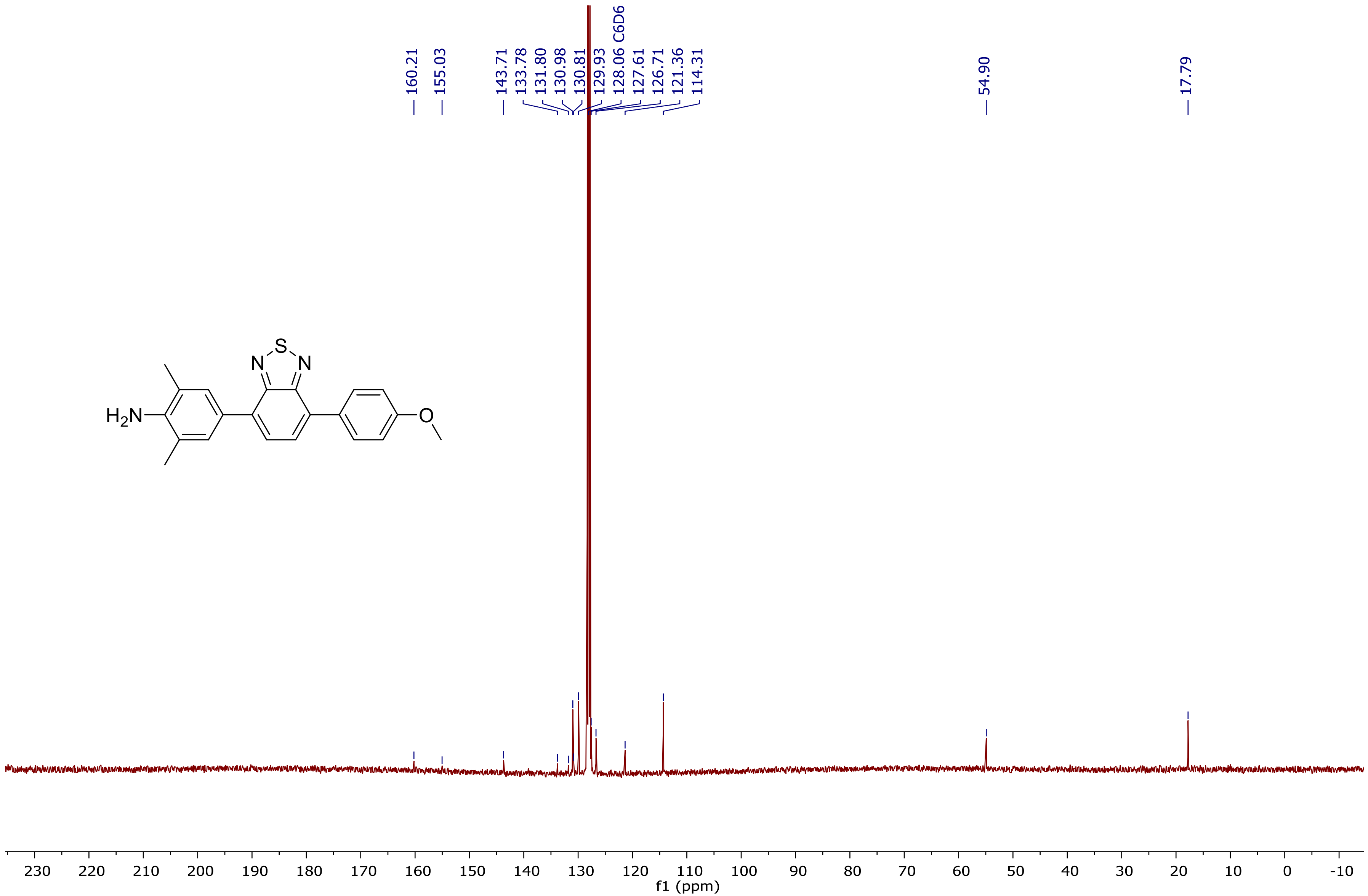

Figure S14. **^{13}C NMR (101 MHz, Benzene-*d*$_6$) of 4-(7-(4-methoxyphenyl)benzo[c][1,2,5]thiadiazol-4-yl)-2,6-dimethylaniline (2)**

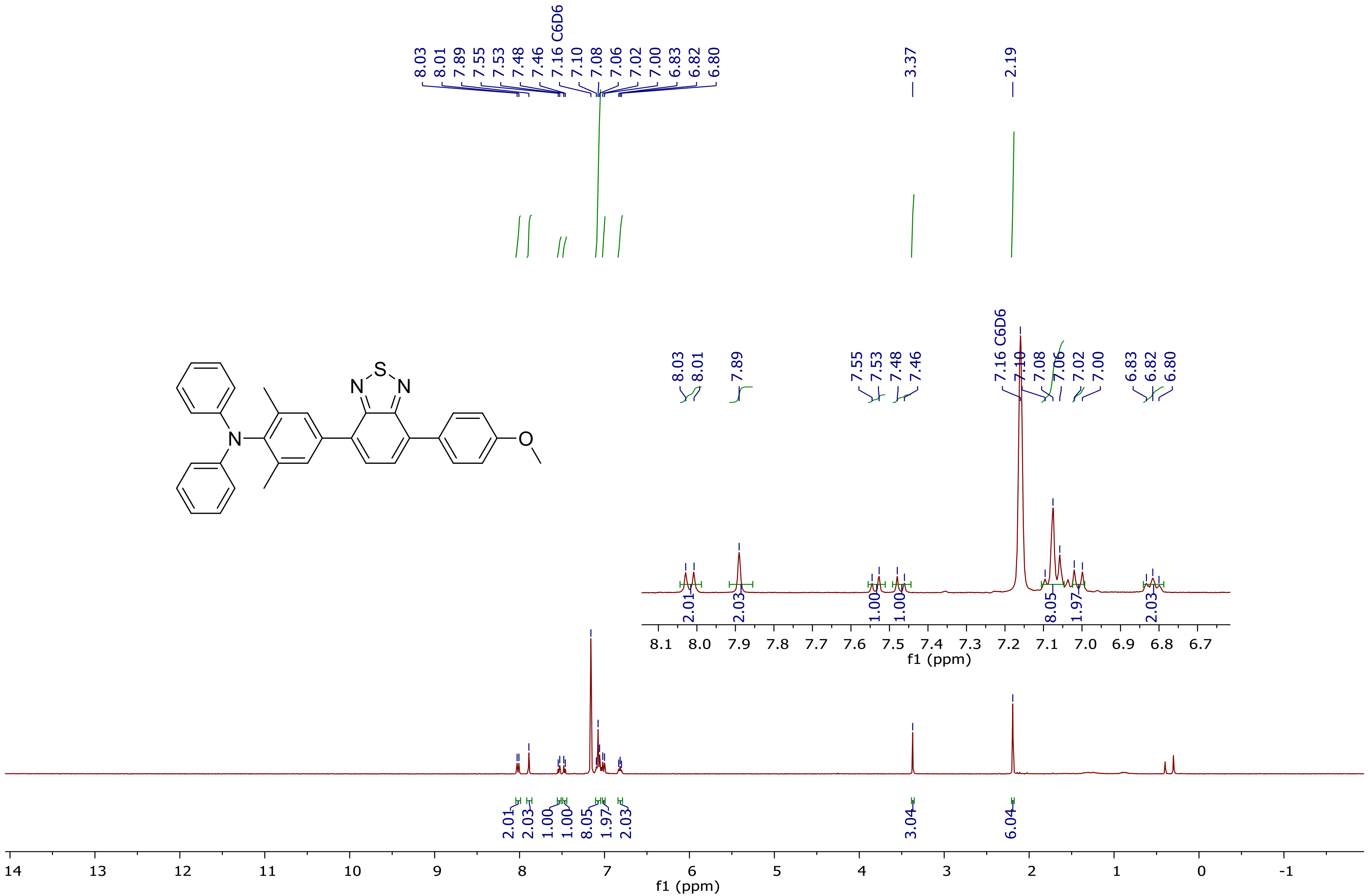

Figure S15. [1]**H NMR (400 MHz, Benzene-*d*$_6$) of 4-(7-(4-methoxyphenyl)benzo[c][1,2,5]thiadiazol-4-yl)-2,6-dimethyl-N,N-diphenylaniline (D1)**

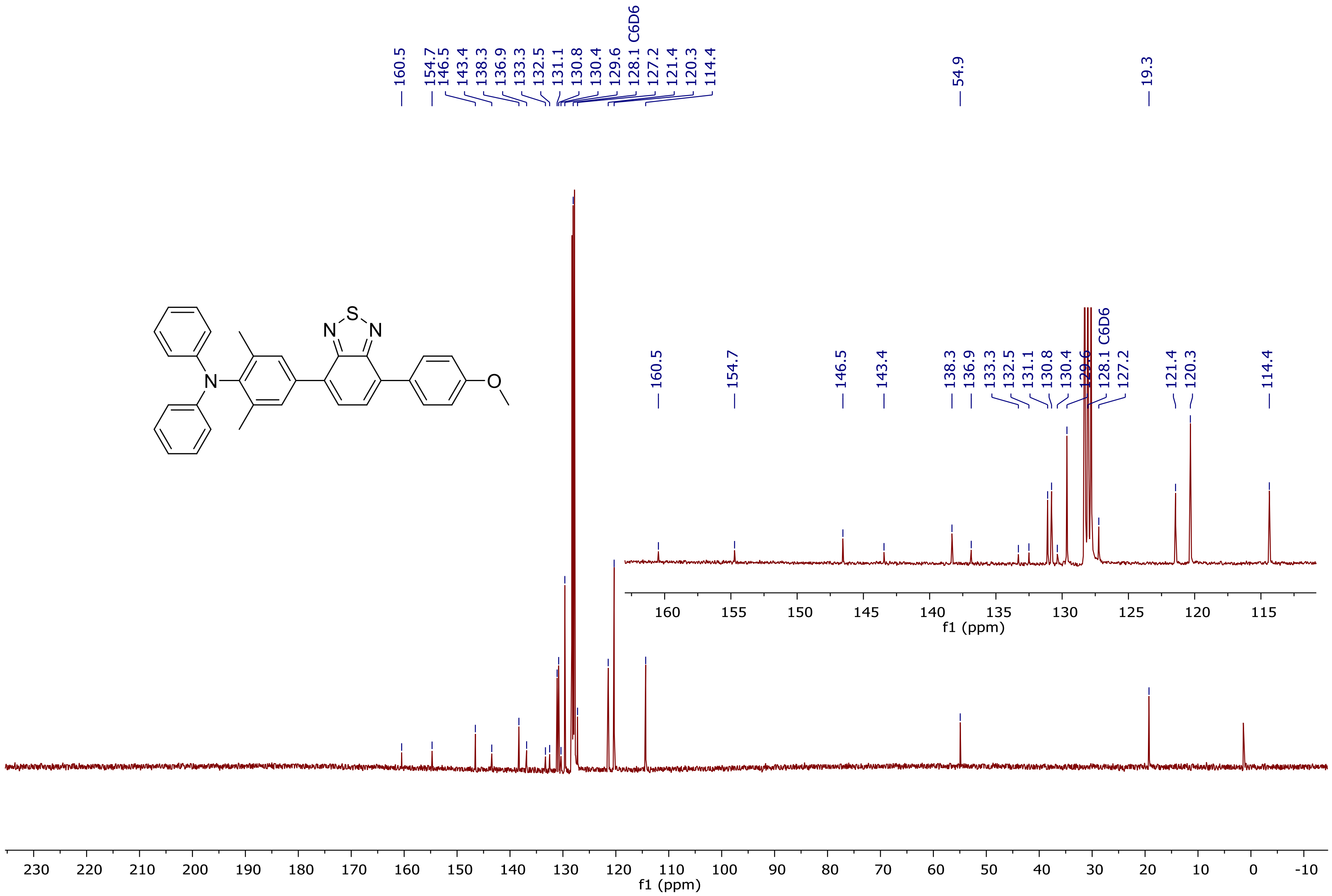

Figure S16. ^{13}**C NMR (101 MHz, Benzene-*d*$_6$) of 4-(7-(4-methoxyphenyl)benzo[c][1,2,5]thiadiazol-4-yl)-2,6-dimethyl-N,N-diphenylaniline (D1)**

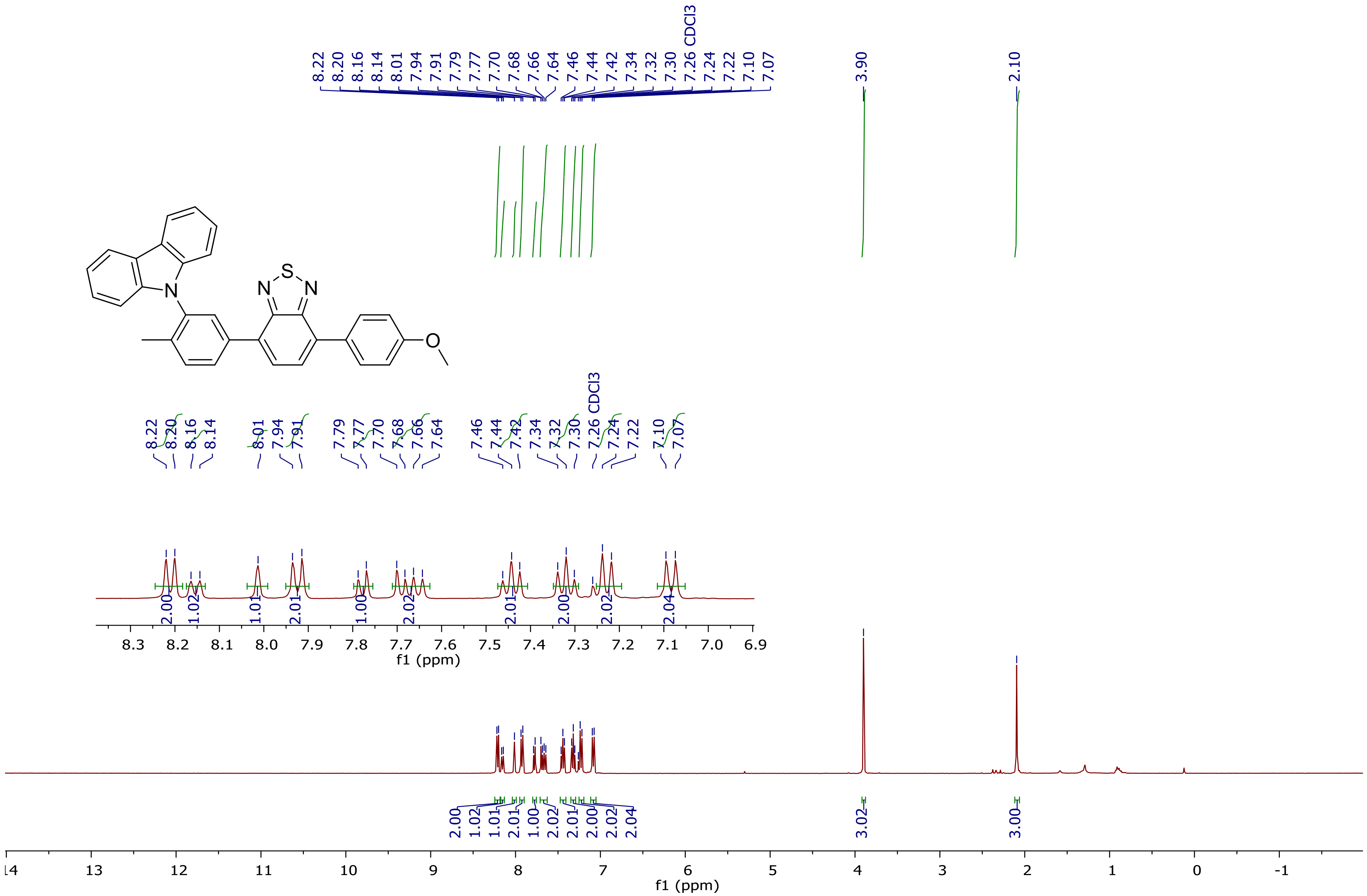

Figure S17. ^{1}H **(400 MHz, Chloroform-*d*) of 4-(3-(9H-carbazol-9-yl)-4-methylphenyl)-7-(4-methoxyphenyl)benzo[c][1,2,5]thiadiazole (D2)**

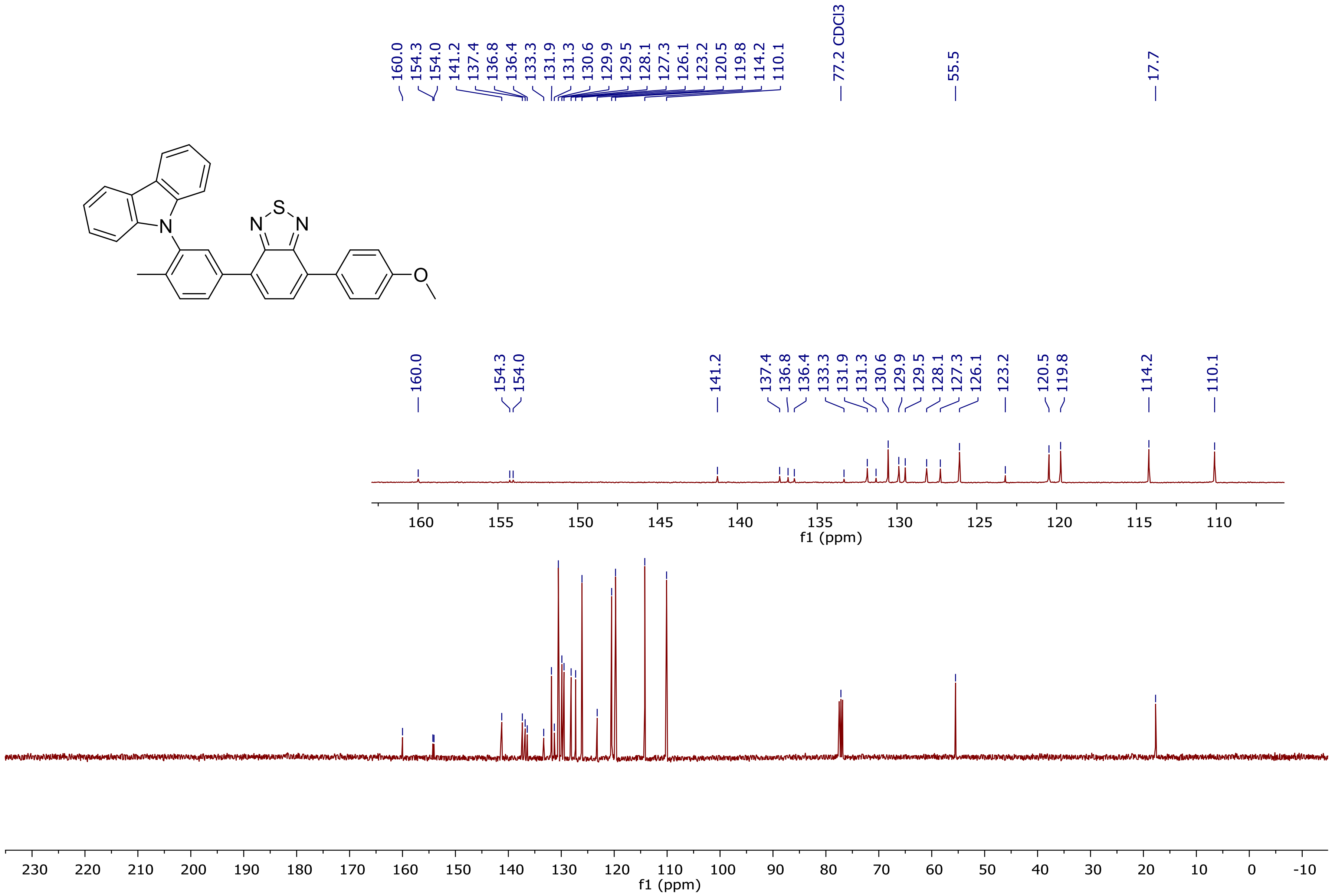

Figure S18. $^{13}C\{^{1}H\}$ **NMR of 4-(3-(9H-carbazol-9-yl)-4-methylphenyl)-7-(4-methoxyphenyl)benzo[c][1,2,5]thiadiazole (D2)**

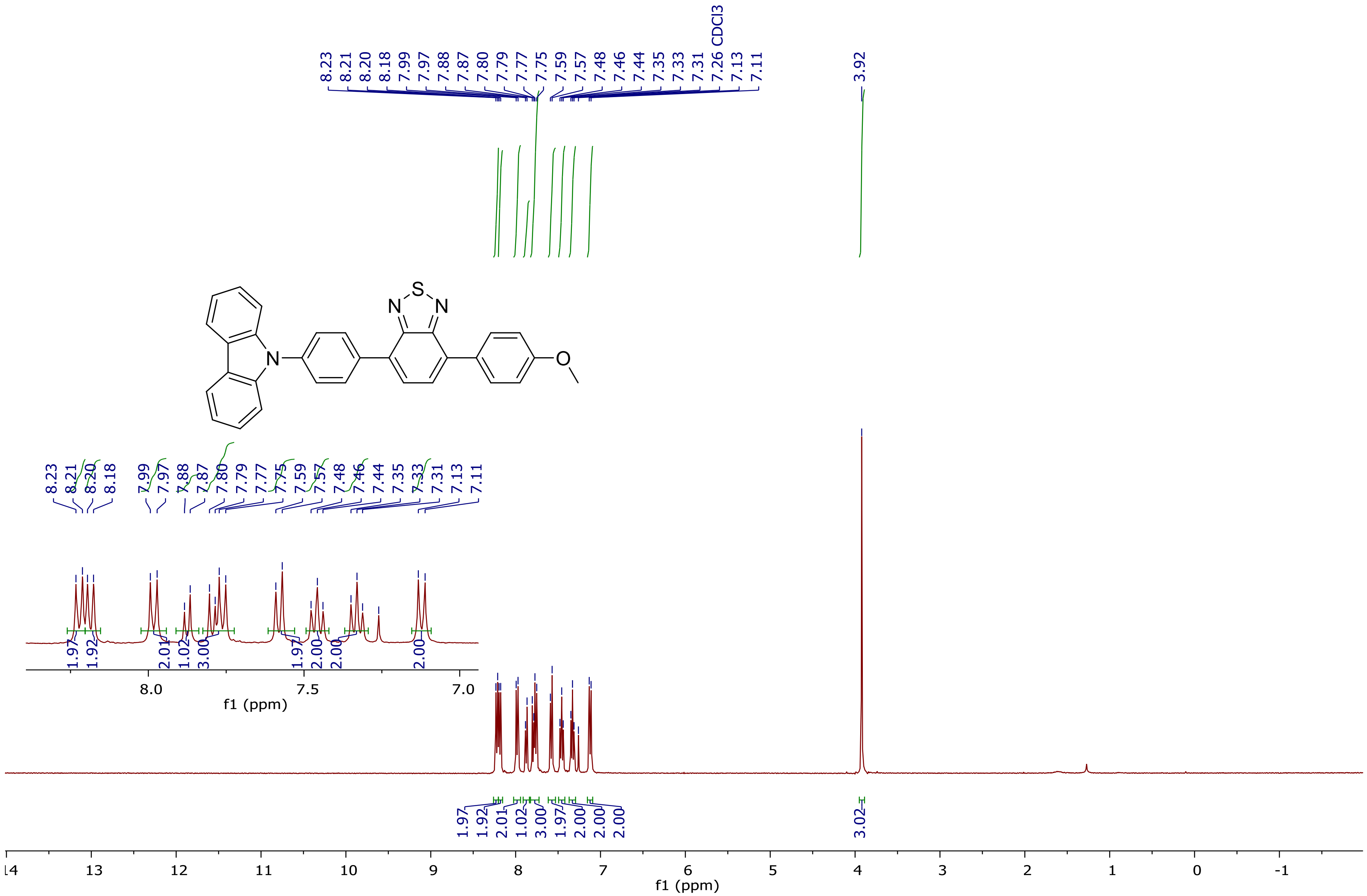

Figure S19. ^{1}**H (400 MHz, Chloroform-*d*) of 4-(4-(9H-carbazol-9-yl)phenyl)-7-(4-methoxyphenyl)benzo[c][1,2,5]thiadiazole (D3)**

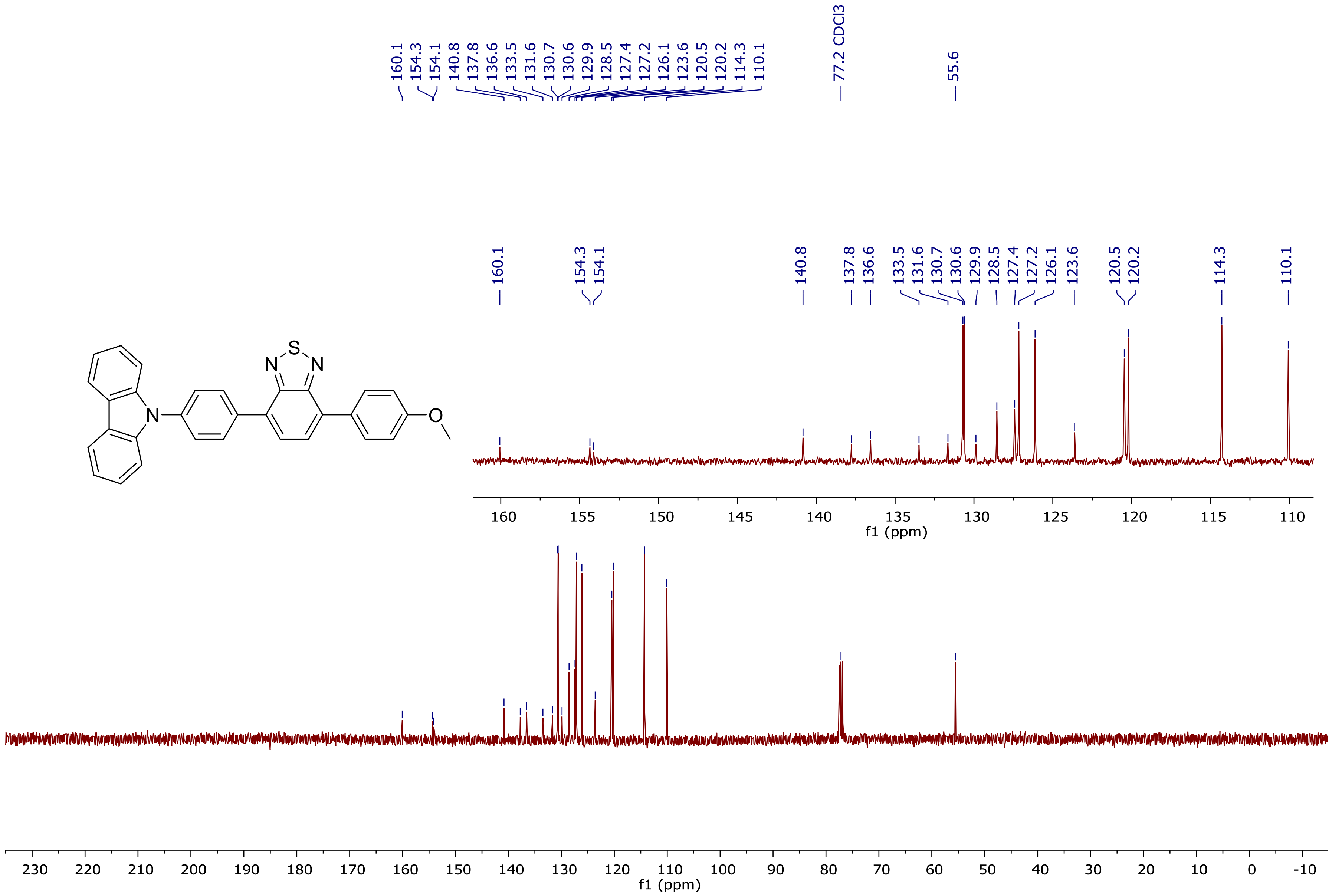

Figure S20. $^{13}C\{^{1}H\}$ **NMR of 4-(4-(9H-carbazol-9-yl)phenyl)-7-(4-methoxyphenyl)benzo[c][1,2,5]thiadiazole (D3)**
